# Transcriptional Regulation of Quinoa Seed Quality: Identification of Novel Candidate Genetic Markers for Increased Protein Content

**DOI:** 10.3389/fpls.2022.816425

**Published:** 2022-06-02

**Authors:** Åsa Grimberg, Ganapathi Varma Saripella, Ritva Ann-Mari Repo-Carrasco Valencia, Therése Bengtsson, Gabriela Alandia, Anders S. Carlsson

**Affiliations:** ^1^Department of Plant Breeding, Swedish University of Agricultural Sciences, Alnarp, Sweden; ^2^CIINCA, Universidad Nacional Agraria La Molina (UNALM), Lima, Peru; ^3^Department of Plant and Environmental Sciences, University of Copenhagen, Copenhagen, Denmark

**Keywords:** plant protein, RNA sequencing, SNP, transcription factor, transcriptional regulation, transcriptome-based markers, *Chenopodium quinoa*

## Abstract

Quinoa (*Chenopodium quinoa* Willd.) is a crop that has great potential for increased cultivation in diverse climate regions. The seed protein quality obtained from this crop is high concerning the requirements to meet human nutritional needs, but the seed protein content is relatively low if compared to crops such as grain legumes. Increased seed protein content is desirable for increasing the economic viability of this crop in order for it to be used as a protein crop. In this study, we characterized three genotypes of quinoa with different levels of seed protein content. By performing RNA sequencing of developing seeds, we determined the genotype differences in gene expression and identified genetic polymorphisms that could be associated with increased protein content. Storage nutrient analyses of seeds of three quinoa genotypes (Titicaca, Pasankalla, and Regalona) from different ecoregions grown under controlled climate conditions showed that Pasankalla had the highest protein content (20%) and the lowest starch content (46%). Our seed transcriptome analyses revealed highly differentially expressed transcripts (DETs) in Pasankalla as compared to the other genotypes. These DETs encoded functions in sugar transport, starch and protein synthesis, genes regulating embryo size, and seed transcription factors. We selected 60 genes that encode functions in the central carbon metabolism and transcription factors as potential targets for the development of high-precision markers. Genetic polymorphisms, such as single nucleotide polymorphisms (SNPs) and base insertions and deletions (InDels), were found in 19 of the 60 selected genes, which can be further evaluated for the development of genetic markers for high seed protein content in quinoa. Increased cultivation of quinoa can contribute to a more diversified agriculture and support the plant protein diet shift. The identification of quinoa genotypes with contrasting seed quality can help establish a model system that can be used for the identification of precise breeding targets to improve the seed quality of quinoa. The data presented in this study based on nutrient and transcriptome analyses contribute to an enhanced understanding of the genetic regulation of seed quality traits in quinoa and suggest high-precision candidate markers for such traits.

## Introduction

### Quinoa as a Protein Crop

Quinoa (*Chenopodium quinoa* Willd. 2*n* = 4x = 36) is an ancient crop that is still grown today mostly in South America, primarily for food purposes, and is consumed as a cereal grain (Tapia et al., 1979; Palomino et al., 2008; Alandia et al., 2020). Although regarded as a minor crop, it has received interest worldwide as a highly nutritious and delicious food crop that can be grown in harsh environments, such as in poor and saline soils in dry climates (Ruiz et al., 2014; Hena et al., 2016; Rasmussen et al., 2016; López-Marqués et al., 2020). This increased attention is reflected by the total harvested area of quinoa, which has increased fourfold globally from around 50,000 ha in the 1960’s up to 200,000 ha today (Alandia et al., 2020; FAO, 2022). The Food and Agriculture Organization declared 2013 as “the international year of quinoa” to highlight the importance of this crop with regard to food security and agrobiodiversity (Home-International Year of Quinoa, 2013). In Europe, the high dependence on imported soybean (*Glycine max*) to meet the increasing needs for plant proteins has led to attempts to expand the cultivation of different protein crops (Development of plant proteins in the EU, 2018). Among these protein crops, quinoa has significant potential to be cultivated on marginal lands and, thereby, contribute to the transition toward more resilient and sustainable food systems. The current cultivated quinoa germplasms exhibit high genetic diversity and adaptation to a wide range of environments (Ruiz et al., 2014), which gives promise for the development of new varieties that will be suitable for different regions in the world. For example, quinoa germplasm adapted to tropical highland environments, such as the Andes region in South America, can be suitable for the temperature requirements in Northern Europe (Christiansen et al., 2010). However, there can be large differences in day length between these environments, and germplasm adapted to coastal conditions in Chile has been important for the development of material with early ripening under Northern European conditions (Jacobsen, 2017).

### Seed Quality Is an Important Plant Breeding Target

Another aspect to take into account from a breeding perspective on quinoa is to exploit variation in seed quality in available germplasm. The allocation of carbon between different storage compounds, typically starch, protein, and oil, and the quality of these compounds in seeds from different crops determine seed quality and, thereby, their end uses. One reason to why seeds from different plant species have different nutrient composition is due their differences in seed morphology (Baud and Lepiniec, 2010). For example, oil crop seeds usually have a high embryo proportion, which is a tissue that is generally dense in oil, while cereal grains usually have a high endosperm proportion, which is usually dense in starch (Baud and Lepiniec, 2010). However, differences in the proportions of the different seed tissues, and their chemical composition, can also be found in different varieties of the same crop such as for oats in which both high- and low-oil varieties are available (Banas et al., 2007). In quinoa, the protein and oil are mostly stored in the embryo, while starch is mostly deposited in the perisperm (a tissue that originates from the non-fertilized nucellus in contrast to the triploid endosperm in cereals) which constitutes the majority of the seed tissues (Burrieza et al., 2014). The protein content in quinoa seeds ranges between 12% and 23% by dry weight (dw) in different varieties (Dakhili et al., 2019), which is higher than that in cereals (10%–15% by dw) but lower than that in other major protein crops such as soybean (45% by dw). The protein quality in quinoa is high, with an amino acid composition that is particularly rich in lysine, histidine, and methionine, which, from a human nutritional perspective, is superior to that of the common cereals (Dakhili et al., 2019). Due to the high protein quality, there is an increased interest in developing nutritious food products based on protein extracts or isolates obtained from quinoa seeds. Therefore, developing quinoa varieties with increased seed protein content is desirable for increasing the protein yields, which would benefit the entire value chain of this crop.

### Genetic Regulation of Seed Protein Content

Seed quality is determined by both genetic and environmental factors. In one study under Mediterranean climate conditions, the protein content of quinoa seeds was shown to be positively correlated to both yield and nitrogen fertilization levels (Geren, 2015), which is not the case in wheat grains (Kramer, 1979; Zörb et al., 2018). By studying genotypes with contrasting seed quality, we aim to identify genetic factors that regulate protein content that can become plant breeding targets for developing new, improved varieties with increased protein content in the grain. In a recently published study, we compared three quinoa genotypes of diverse origins that were identified to have contrasting seed protein content, and, through X-ray micro-tomography, a positive correlation was found between protein content and embryo proportion (Gargiulo et al., 2019). This is not surprising if we consider that the majority of the protein in quinoa seeds is, in fact, deposited in the embryo tissues (Burrieza et al., 2014). These results prompted us to further examine these genotypes to enhance our understanding of the genetic factors that regulate protein content in quinoa seeds and how this can help identify genetic markers that can be used for breeding improved quinoa varieties.

### Development of High-Precision Genetic Markers for Quinoa Seed Quality

Among different types of genetic markers, single nucleotide polymorphisms (SNPs) have gained popularity mainly due to their high abundance in genomes and the increased availability of efficient low-cost nucleotide sequencing technologies (Kim et al., 2020). The complete genome of quinoa was sequenced in 2017 (Jarvis et al., 2017), which enables new possibilities for developing genomic breeding tools for this crop (Alandia et al., 2021). However, the genetic and molecular understanding of specific target traits for quinoa is needed for the strategic development of improved varieties.

SNP markers for desired traits for a crop can be identified by genotyping several hundreds of genotypes (through DNA-sequencing) that can statistically be associated to phenotyping data in genome-wide association studies (GWAS; Tibbs Cortes et al., 2020). Another approach to identify SNP markers in plants is to focus the analysis on the gene-based regions of the genome by using transcriptome analysis (cDNA sequencing of isolated mRNA) from specific plant tissues of a few genotypes (Kaur et al., 2012; Parra-González et al., 2012; Mantello et al., 2014; Webb et al., 2016; Nie et al., 2017; Tsehay et al., 2020) which can also reduce the complexity of genomes. Genetic polymorphisms, such as SNPs and base insertions and deletions (InDels), which can all be called short variants, between the genotypes can be identified in target genes that are likely to affect the function of the gene product (such as a protein involved in a metabolic pathway) and, thereby, also affect the phenotype. It should be noted that short variants found through transcriptome-based analyses can also affect the function of the encoded protein by causing alternative splicing of mRNA (Luo et al., 2017). Markers have been developed based on transcriptome analyses for many crops (Kaur et al., 2012; Parra-González et al., 2012; Mantello et al., 2014; Webb et al., 2016; Nie et al., 2017; Tsehay et al., 2020) but not often fully exploiting the possibility of identifying interesting target genes through genotype comparisons.

Transcriptome-based analyses of quinoa have been made to identify target genes and markers for drought and stress tolerance (Raney et al., 2014; Morales et al., 2017; Schmöckel et al., 2017) and also recently to characterize genetic differences in seed quality concerning flavonoid and proanthocyanin levels (Wang et al., 2020; Liu et al., 2022). However, knowledge about the particular genetic factors that regulate seed protein content in quinoa is lacking. In this study, we carried out a comparative transcriptome analysis based on RNA sequencing for developing quinoa seeds from three genotypes with diverse seed quality. We determined genotype differences in gene expression and identified 60 genes that are encoding functions in the central carbon metabolism and transcription factors as potential gene targets for the development of high-precision genetic markers. Furthermore, SNPs/InDels were identified in these genes, which can be used for the further evaluation of genetic markers for increased protein content in quinoa.

## Materials and Methods

### Plant Material, Growth Conditions, and Seed Sampling

Commercially available quinoa cultivars Titicaca (Quinoa Quality, Regstrup, Denmark) and Regalona Baer (Semillas Baer, Temuco, Chile) and the landrace Pasankalla rosada obtained from Universidad Nacional del Altiplano in Peru (facilitated by the University of Copenhagen) were grown in 3.5 L pots growth chambers (Biotrone, SLU-Alnarp) under fluorescent light (200 μmol·m^−2^ s^−1^) using a 12/12 h (light/dark) photoperiod with temperatures of 20°C/17°C and 70% humidity. The top panicle of each plant was labelled by anthesis. All varieties started to flower 32–42 days after germination. Seeds from three biological replicates were sampled at three different developmental stages (called early, mid, and late) and put on ice, sepals were then removed using forceps and seeds were put in a plastic tube in N_2_ (L) until reaching a volume of 2–3 ml seeds and was then stored at −80°C. The early, mid, and late stages represented 15, 25, and 35 days post anthesis (dpa) for Titicaca and Regalona, respectively. Seeds of Pasankalla started to develop later after anthesis, resulting in corresponding time points of approximately 28, 40, and 52 dpa. Seeds were also harvested at maturity and stored at room temperature. The sampling of seeds was always done at approximately the same time of day (4–6 h into the light period) to avoid any potential differences due to diurnal fluctuations in gene expression and nutrient levels. Seed samples were ground in 35 ml steel containers using steel beads (16 mm in diameter) and were chilled in liquid nitrogen to prevent them from thawing at any time using a mixer mill (Retsch, Haan, Germany) at 30 Hz for 2 × 15 s. Flour was stored at −80°C until further analyses. An aliquot of this flour was freeze dried for 48 h prior to metabolite analyses. Three biological replicates (i.e., seeds from three individual plants) were used for all analyses.

### Seed Composition Analyses

Protein content was determined as total N x 6.25, where total N in freeze dried flour was determined with an elemental analyzer (Flash 2000, ThermoScientific). In brief, amounts of 2 ± 0.1 mg of freeze dried flour were weighed into tin capsules (Mettler, Toledo XP6), which were then folded and analyzed as described previously (Grimberg et al., 2020). Amino acid composition of freeze dried flour from mature seeds was determined using ion exchange chromatography according to the method described in EU 152/2009 (Eurofins Food and Feed Testing, Lidköping, Sweden). Starch content of freeze dried flour (50 mg) was determined using the Total Starch Determination Kit (Megazyme, Wicklow, Ireland), and soluble sugars were first removed using 80% aqueous EtOH. Oil content was determined as previously described (Grimberg et al., 2018) except that fatty acid methyl esters (FAMEs) were separated on a DB-23 column (30 m, inner diameter 0.25 mm, film thickness 0.25 μm). In brief, lipids from total lipid extracts from flour were separated using thin layer chromatography to isolate triacylglycerol (oil). The triacylglycerol was then scraped from the plates and further methylated into FAMEs, which were separated using a gas chromatograph split into a mass spectrometer for identifying the FAMEs and a flame ionization detector for quantification of the FAMEs (GCMSD5977 with Intuvo GC, Agilent, Santa Clara, United States). Oil content was calculated as the total weight of all fatty acid methyl esters in triacylglycerol. Total phenolic compounds, flavonoids, and antioxidant activity was analysed on 200 mg flour as previously described (Singleton and Rossi, 1965; Dini et al., 2010; Hirose et al., 2010). In brief, compounds were extracted by mixing the flour with aqueous methanol (for total phenolic compounds and flavonoids) or pure methanol (for antioxidant analysis), then left for 24 h in darkness and centrifuged followed by spectrophotometric analysis. Total phenolic content was determined as gallic acid equivalents after reaction with Folin-Ciocalteau reagent, flavonoid content as catechin equivalents after mixing with sodium nitrite, aluminium chloride and sodium hydroxide, and, finally, antioxidant activity as amount of trolox after reaction with 2,2-diphenyl-1-picrylhydrazyl. All seed composition analyses were made on three biological replicates. Data were statistically analyzed using ANOVA followed by Fisher’s or Tukey’s test (*p* < 0.05, LSD) to compare means of treatment groups (Minitab 18).

### RNA Extraction and Sequencing

Total RNA was extracted from ground flour from three biological replicates using PureLink Plant RNA Reagent (Ambion, Foster City, United States) and then stored in −80°C. Due to difficulties to get high enough RNA quality of seeds from the late developmental stage, only RNA from the early and mid-stages were used further. Total RNA was DNase-treated (Turbo DNase, Ambion), and the integrity of RNA was then determined using Experion RNA StdSens analysis kit (BioRad, Hercules, United States) before diluting to 50 ng/μl. The cDNA libraries were prepared through polyA selection, and paired-end reads were generated using Illumina HiSeq sequencing (Eurofins Genomics, Ebersberg, Germany).

### RNA Sequencing Data Analysis and Gene Annotation

Paired-end mRNA reads (2 × 150 bp) were processed with an initial quality control (QC) check using FastQC v0.11.7 (Andrews, 2010), all types of ribosomal RNAs (rRNAs) were removed using Sortmerna-v2.1b (Kopylova et al., 2012), and adapters were trimmed with Trimmomatic-v0.36 (Bolger et al., 2014) using a sliding window of 5:20, with a minimum length of 20 and using default values for the other settings. Technical replicate libraries were merged, and multi-sample QC reports were generated with MultiQC v1.6 tool (Ewels et al., 2016). Splice aligner STAR-v2.5.4a (Dobin and Gingeras, 2015) was used with default settings and the *twopassMode Basic* option to align all sample (3 × 6 = 18) reads to the whole genome of *C. quinoa* (cultivar: QQ74, ASM168347v1). Transcript level read quantification was performed using Salmon v1.3.0 (Patro et al., 2017). Minimum quality criteria were used to remove lowly expressed genes (retained genes with counts-per-million (cpm) greater than one in at least two samples). Raw read counts were used for differential expression (DE) analysis with DESeq2 (Anders and Huber, 2010; Anders et al., 2013) and in-built cross sample Relative Log Expression (RLE; Love et al., 2014) normalization was performed.

Despite the availability of annotation from the quinoa reference genome, we have performed additional annotation by performing Blast searches (ncbi-blast-v2.9.0) of transcripts against available transcript databases from *Arabidopsis thaliana* (TAIR10.1) and *C. quinoa* Willd v1.0 (Jarvis et al., 2017) to obtain more information about the function of homologous genes. Moreover, functional annotations of genes from transcription factor databases such as PlantTFDBv5.0^1^ and ITAKv18.12^2^ were included.

The Gene Ontology (GO) and Kyoto Encyclopedia of Genes and Genomes (KEGG) enrichment analysis were performed on the list of genes identified to be differentially expressed in Pasankalla as compared to the other two genotypes, by using obtained gene coordinates from *Arabidopsis thaliana* (TAIR10.1). GO over-representation test was performed with enrichGO^3^ submodule from the clusterProfiler (v3.18.1)^4^ R-package with settings pvalueCutoff = 0.01, qvalueCutoff = 0.01 and keeping remaining settings as default. The KEGG Pathway enrichment analysis was performed with Gene Set Enrichment Analysis (GSEA) of KEGG tested with module gseKEGG^5^ with settings nPerm = 10,000, value of *p* cutoff = 0.01.

### SNP Calling

We employed independent sample variant calling (SNP and InDels) approach for the three genotypes by concatenated biological replicates for each genotype. For this, the GenomeAnalysisTK (GATK_v3.8.1)^6^ HaplotypeCaller was used with best-practice user’s pipeline. Annotation of the variants was processed using the SnpEff v4.3t tool^7^ to predict the effect of gene variants on genes and proteins.^8^ The workflow scripts are available in the Github repository, https://github.com/gvarmaslu/RNAseq_Quinoa-seed.

## Results

### Genotype Differences in Seed Composition

**Figure 1:**
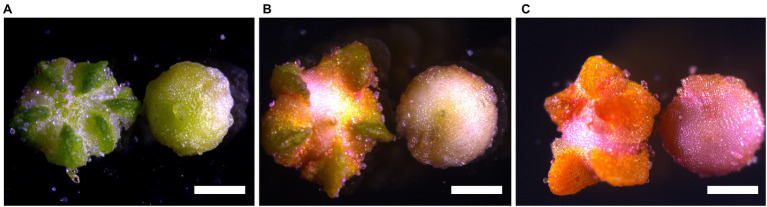
Microscopy photos of quinoa seeds (Titicaca) at the early **(A)**, mid **(B)**, and late **(C)** developmental stages used in this study. These developmental stages correspond to the following days post anthesis (dpa) for Titicaca: 15 dpa (early), 25 dpa (mid), and 35 dpa (late). Sepals have been removed from seeds on the right in each panel. Scale bars are 1.07 mm.

To characterize nutrient composition during seed development, we cultivated three quinoa genotypes under controlled environment and sampled the seeds at different developmental stages. The protein content of these seeds at maturity was previously shown to be different for these genotypes (Gargiulo et al., 2019). The three selected quinoa genotypes, i.e., Titicaca, Pasankalla rosada (henceforth called Pasankalla) and Regalona Baer (henceforth called Regalona), are from different ecoregions and, therefore, have different day-length sensitivities. Pasankalla is a landrace from the highlands type (Adolf et al., 2012; Murphy et al., 2018), Regalona is a cultivar of the coastal type from Chile (Von Baer et al., 2009) and Titicaca is a cultivar developed from coastal landraces in Chile (Adolf et al., 2012). Although the selected day length (12 h) in the controlled climate chambers allowed for flower induction in all genotypes at approximately the same time (35–42 days after germination), the seed development in Pasankalla was delayed as compared to the other genotypes. Therefore, while the seeds of Titicaca and Regalona were sampled at 15, 25, and 35 dpa, the sampling of Pasankalla was adjusted based on visual inspection of the seeds to determine comparable time points, which occurred at approximately 28, 40, and 52 dpa, respectively. Photos of the different developmental stages of the quinoa seeds are presented in Figure 1. The dry matter content of the seeds [% by fresh weight (fw)] increased during development, as expected, and was approximately the same for all three genotypes at each sampled developmental stage (Supplementary Figure S1). This confirmed that the adjusted time points for the sampling of the Pasankalla seeds were comparable to the developmental stages that were sampled of Titicaca and Regalona, and for simplicity the stages are henceforth called mid, early, and late.

**Figure 2:**
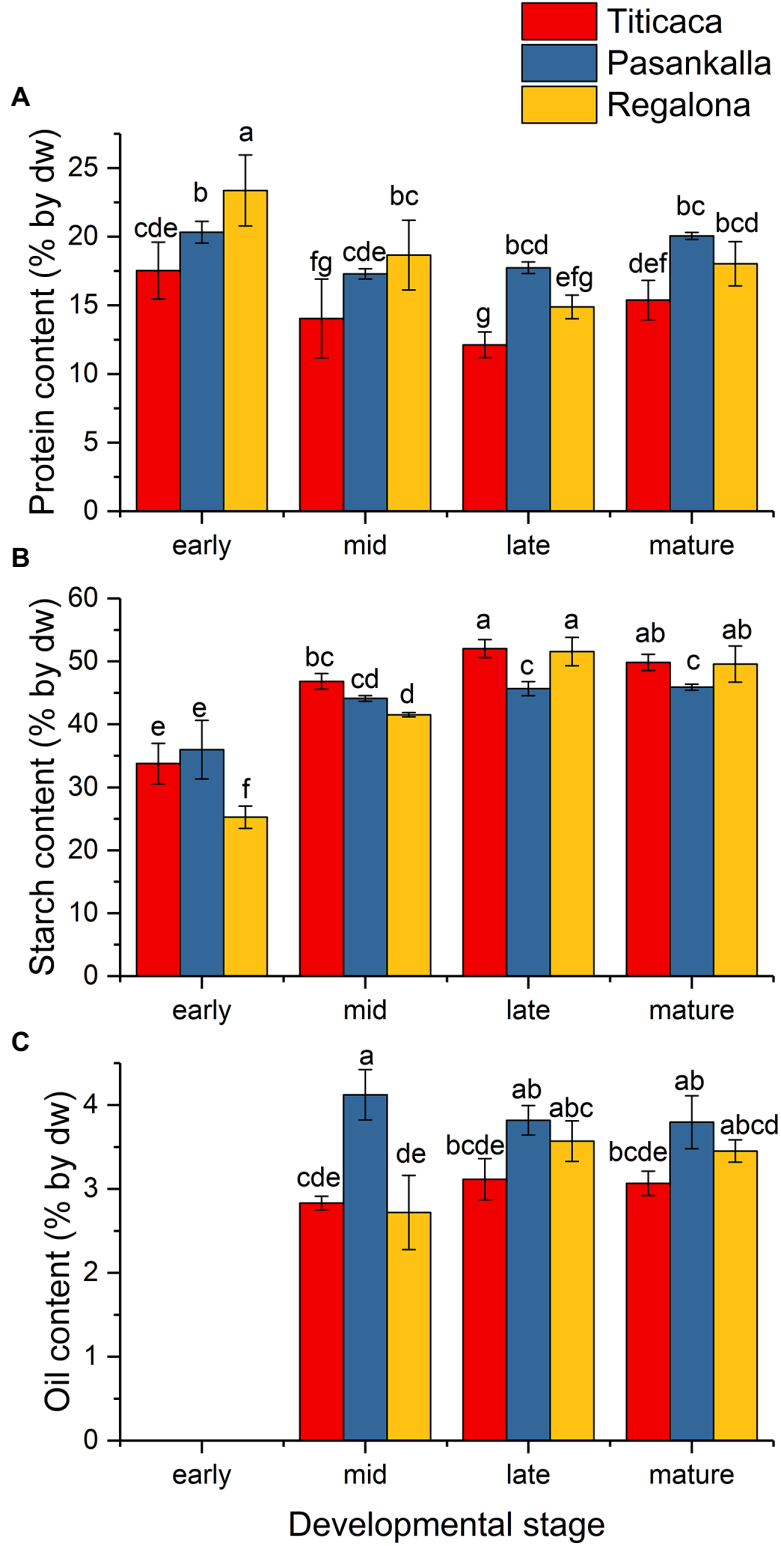
Protein **(A)**, starch **(B)**, and oil **(C)** content [% by dry weight (dw)] in developing seeds of quinoa Titicaca, Pasankalla, and Regalona. The protein content in mature seeds of these genotypes was published previously (Gargiulo et al., 2019). Plants were grown under controlled growth conditions. For corresponding dpa for each developmental stage, see the text. Results are showing mean values ± SD of three biological replicates. Bars that do not share a letter are significantly different according to Fisher’s pairwise test (*p* ≤ 0.05).

**Figure 3:**
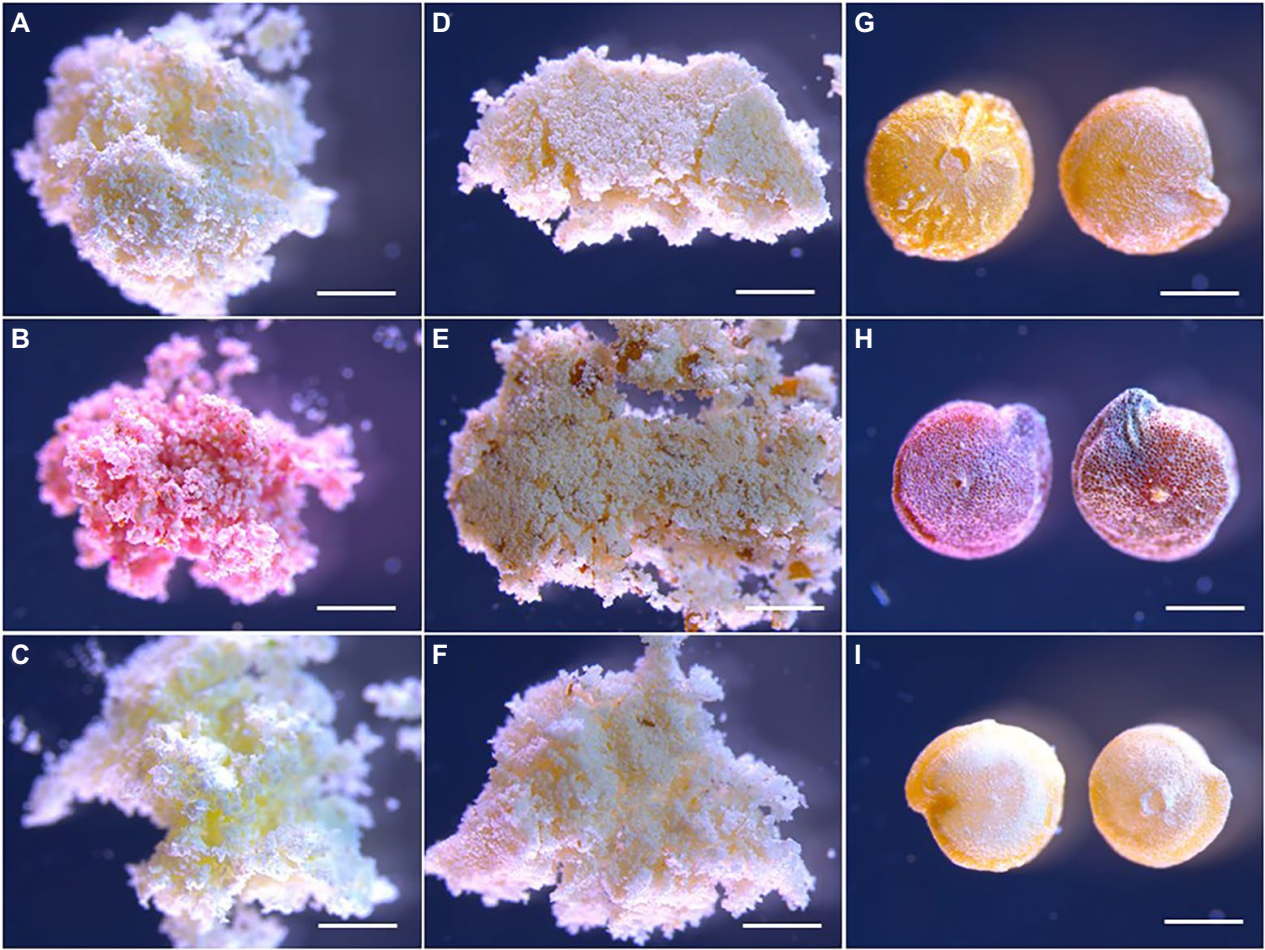
Light microscopy photos of quinoa seed flour from the mid developmental stage **(A–C)**, flour at maturity **(D–F)** and whole seeds at maturity **(G-I)** of Titicaca **(A,D,G)**, Pasankalla **(B,E,H)**, and Regalona **(C,F,I)**. For corresponding dpa for the mid developmental stage, see the text. Scale bars are 1 mm.

The content of the major storage compounds was determined for freeze-dried flour prepared from seeds of the studied quinoa genotypes at the different developmental stages. Storage nutrient analyses revealed genotype differences in protein, starch, and oil content of the seeds on a dw basis (Figure 2). Pasankalla was found to have the highest protein content (20%) and the lowest starch content (46%) at maturity. Further, the oil content of the Pasankalla seeds was higher than that of the other genotypes throughout seed development, with an oil content of 3.8% in mature seeds. However, this was only statistically significant at the mid developmental stage. The lowest protein and oil content was found for Titicaca (15% and 3.1% respectively), which, instead, had a higher starch content (50%). It could be noted that the seeds of Pasankalla most probably contained more pigments than the other genotypes, based on visual inspection of seeds and seed flour at the mid developmental stage although being almost completely absent at maturity (Figure 3). The total amount of phenolic compounds, flavonoids and antioxidant capacity was measured in seed flour and was found to be highest in Pasankalla among the three genotypes (Supplementary Figure S2).

**Figure 4:**
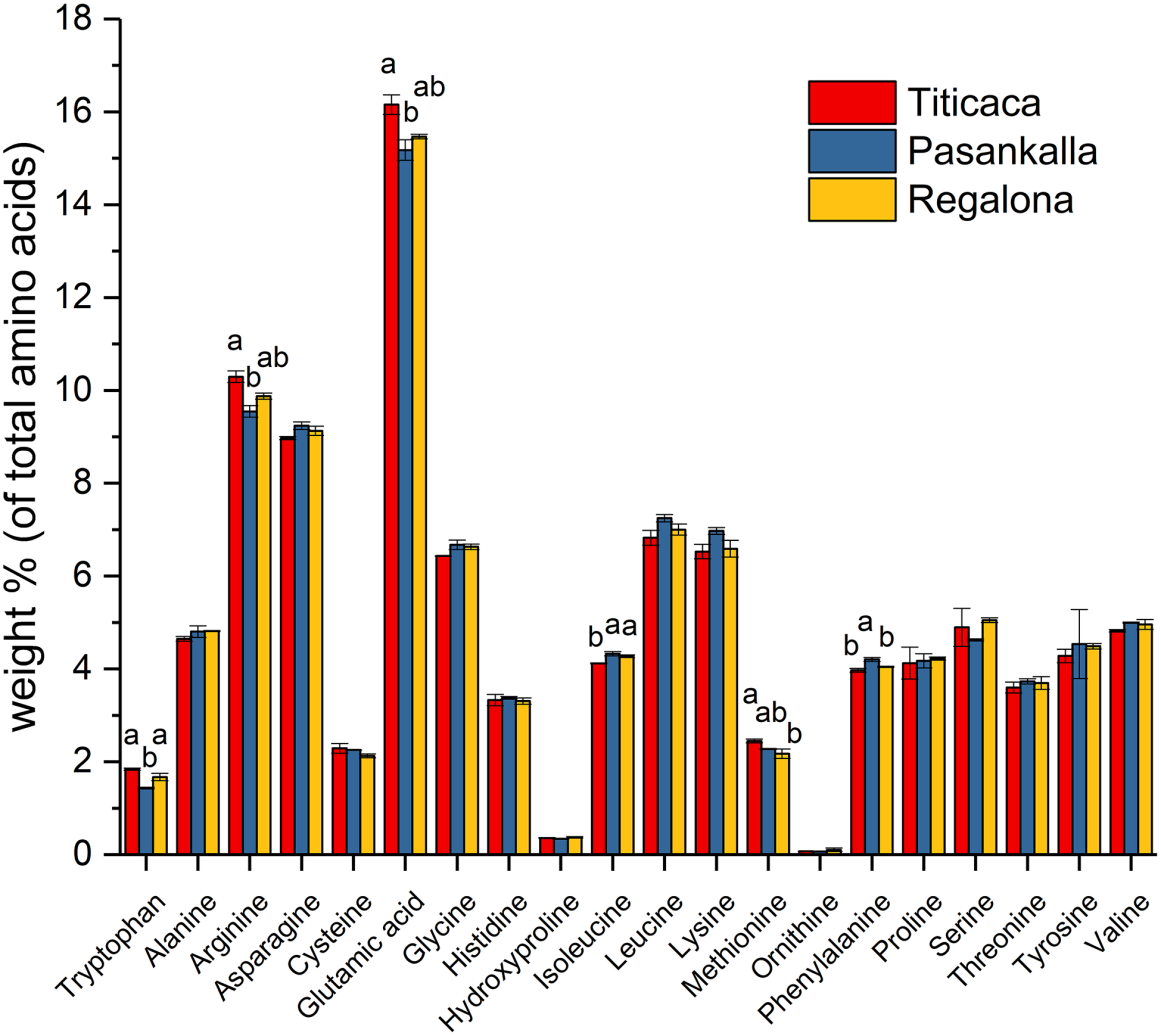
Amino acid profile in mature quinoa seeds of Titicaca, Pasankalla, and Regalona. Results are showing mean values ± SD of two biological replicates. Bars for amino acids showing significant differences between genotypes are marked with letters. Bars that do not share a letter are significantly different according to Tukey’s test (*p* ≤ 0.05).

**Figure 5:**
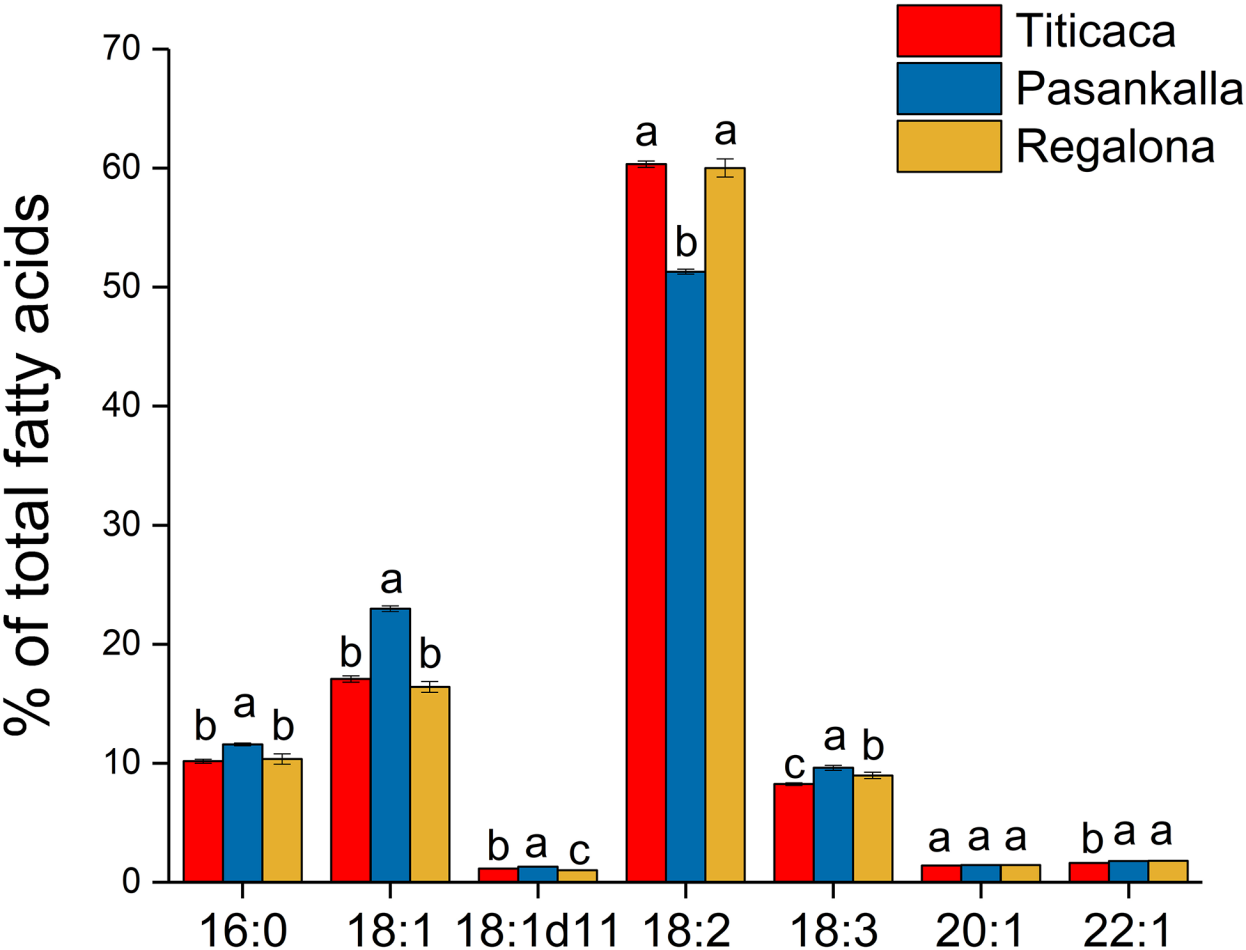
Fatty acid profile (mol % of total fatty acids) of triacylglycerol (oil) in mature quinoa seeds of Titicaca, Pasankalla, and Regalona. Results are showing mean values ± SD of three biological replicates. Bars that do not share a letter (for each individual fatty acid) are significantly different according to Tukey’s test (*p* ≤ 0.05).

The amino acid profile of total protein and the fatty acid profile of triacylglycerol (oil) were determined to see whether there were any differences between the genotypes in terms of protein and oil quality, respectively. The major amino acids were glutamic acid (15.6%), arginine (9.9%), asparagine (9.1%), leucine (7.0%), lysine (6.7%), and glycine (6.6%), as shown in Figure 4. Small but significant differences in tryptophan, arginine, glutamic acid, isoleucine, methionine, and phenylalanine content were found among the genotypes. The oil was composed of the common major fatty acids found in plant seeds with palmitic (16:0), oleic (18:1), linoleic (18:2), and linolenic (18:3) acids comprising more than 90% of all fatty acids detected (Figure 5). Linoleic was the predominate fatty acid, constituting 50%–60% of the fatty acid content. A significant increase in the proportion of oleic acid and a concomitant decrease in linoleic acid were observed in the Pasankalla seed oil as compared to the other genotypes (23% and 17%, and 51% and 60%, respectively).

Altogether, the data from nutrient analyses of developing seeds revealed genotype differences in seed quality, with different proportions of the major seed storage compounds being found and Pasankalla presenting the highest protein content. Therefore, seeds from the three genotypes were further characterized using comparative transcriptome analysis in an attempt to identify the genetic factors that determine seed protein content.

### Comparative Transcriptome Analyses of Quinoa Genotypes

**Figure 6:**
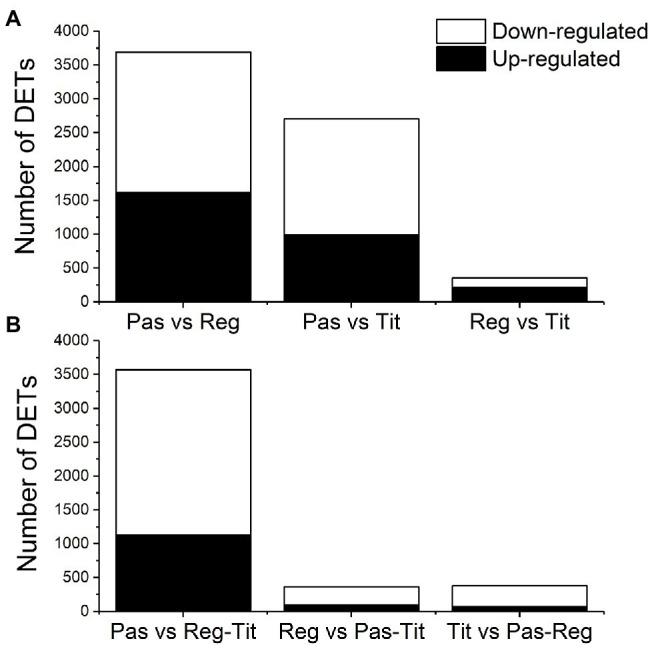
**(A)** Number of differentially expressed transcripts (DETs) as identified by DESeq2 from pairwise comparisons between the three quinoa genotypes Pasankalla, Regalona, and Titicaca after setting thresholds of adjusted value of *p* ≤ 0.01 and log2FC > 1.0. **(B)** Number of differentially expressed transcripts as identified by DESeq2 from comparisons between one genotype against the two other [Pasankalla (Pas), Regalona (Reg), and Titicaca (Tit)] after setting thresholds of adjusted value of *p* ≤ 0.01 and log2FC > 1.0. FC, fold change. Also see data provided in Supplementary Table S6.

**Figure 7:**
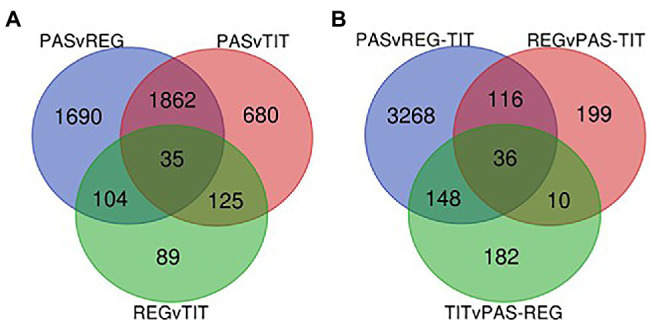
**(A)** Venn-diagram showing the numbers of intersecting genes from the DETs identified from pairwise comparisons of the three genotypes Pasankalla (Pas), Regalona (Reg), and Titicaca (Tit). Adjusted value of *p* ≤ 0.01 and log2FC > 1.0. **(B)** Venn-diagram showing the numbers of intersecting genes from the DETs identified from comparisons of one genotype against the other two genotypes of quinoa. Adjusted value of *p* ≤ 0.01 and log2FC > 1.0. FC, fold change. Also see data provided in Supplementary Table S6.

RNA sequencing was performed on three biological replicates from seed tissues of the three genotypes at two developmental stages (the early and the mid stages) to detect differential gene expression and identify SNPs. The general transcriptome sequence information is found in Supplementary Table S1, which shows that, after quality checks and trimming of the reads, 93%–94% of all sample reads were mapped to the quinoa reference genome that contains a total of 63,173 coding sequences (CDS).^9^ This mapping was used for the estimation of transcript abundance, i.e., for the determination of differentially expressed transcripts (DETs). From preliminary analyses we observed relatively low differences between the early and mid-developmental stages of the same genotype, and therefore we focused our DET analyses on genotype differences only. All three pairwise comparisons between the genotypes showed, by using minimum quality criteria, that around 33,000 transcripts were differentially expressed among the 63,173 protein-coding transcripts available in the reference genome (QQ74). However, by setting more stringent thresholds (adjusted value of *p* ≤ 0.01 and log2 fold change >1), the number of DETs in the pairwise comparisons were reduced to 3,691 in Pasankalla vs. Regalona, 2,702 in Pasankalla vs. Titicaca, and only 353 in Regalona vs. Titicaca (Table 1; Figure 6A). Of the DETs identified in Pasankalla in relation to Regalona or Titicaca, 44% and 37%, respectively, were up-regulated, while the remaining DETs were down-regulated (Figure 6A). An overview of the number of intersecting DETs from the three pairwise comparisons can be seen in the Venn diagram given in Figure 7A. It visualizes that of the 3,691 DETs identified in the comparison between Pasankalla and Regalona (the sum of the numbers in the blue circle in Figure 7A), 1,690 transcripts were differentially expressed between these two genotypes, while 1,862 transcripts were differentially expressed between Pasankalla and Titicaca as well, and with 104 transcripts also being differentially expressed between Regalona and Titicaca. The remaining 35 DETs (the number in the center of the Venn diagram) were the same for all three pairwise comparisons. It should be noted that only 89 DETs were identified as unique to the pairwise comparison between Regalona and Titicaca.

**Table 1:** Intersecting DETs with threshold FDR < 1e-2 and log2FC is set to 1 from pairwise comparisons of quinoa seed transcriptomes between genotypes (one to one; column 2–4, or one to two; column 5–7).

	Pas_vs_Reg	Pas_vs_Tit	Reg_vs_Tit	Pas_vs_Reg-Tit	Reg_vs_Pas-Tit	Tit_vs_Pas-Reg
Number of DETs	3,691	2,702	353	3,568	361	376
Unique genes	3,242	2,320	299	3,072	330	332
AT BLAST-hit genes	2,999	2,076	252	2,776	295	276
TF PlantTFDBv5.0	182	131	12	171	9	11
TF ITAKv18.12	202	144	13	192	11	17

For example, 3,568 transcripts were identified as being differentially expressed in Pasankalla (Pas) as compared to both in Regalona (Reg) and Titicaca (Tit). After removing duplicate transcripts (i.e., representing the same coding strand in the reference genome) on this list it was reduced to 3,072 genes [genes with missing (.) gene information were not included]. Of these 3,072 unique genes 2,776 showed hits in Arabidopsis TAIR database through BLAST searches, and 171 and 192 were annotated as coding for transcription factors as determined from blast searches against two plant transcription factor databases (PlantTFDBv5.0 and TF ITAKv18.12, respectively). For clarity it can also be noted that the sum of all the numbers in for example the blue circle in Figure 7B is 3,568, which can be found in this Table for the Pas_vs_Reg-Tit comparison.

The observation that Pasankalla presented many more DETs in the pairwise comparisons to either Regalona or Titicaca than when comparing Regalona and Titicaca with each other prompted us to explore the comparison of one genotype with the other two. This analysis clearly showed that a much higher number of DETs (3,568) were identified when comparing Pasankalla to both Regalona and Titicaca (the sum of the numbers in the blue circle in Figure 7B) than in the other two comparisons, where only 361 and 376 DETs were identified (Table 1; Figure 6B). Of the DETs identified in Pasankalla compared to both Regalona and Titicaca, 32% were up-regulated, while 68% were down-regulated (Figure 6B). After filtering out the 3,568 DETs in Pasankalla in relation to both Regalona and Titicaca, it was of relevance to take a closer look at the 3,268 DETs found to be unique to this comparison (Figure 7B). The annotation of these transcripts was carried out by identifying corresponding coding strands (CDS) in the quinoa reference genome. If several transcripts or isoforms represented the same gene, the duplicates were removed by selecting a single representative transcript for each gene, which resulted in a slightly lower number of DETs (3,072). Of these transcripts, a majority (2,776) yielded hits in the Arabidopsis genome through BLAST searches of the CDS, which were also included to complement the annotation from the quinoa reference genome. A summary of these numbers and the corresponding numbers for the other genotype comparisons can be found in Table 1.

**Figure 8:**
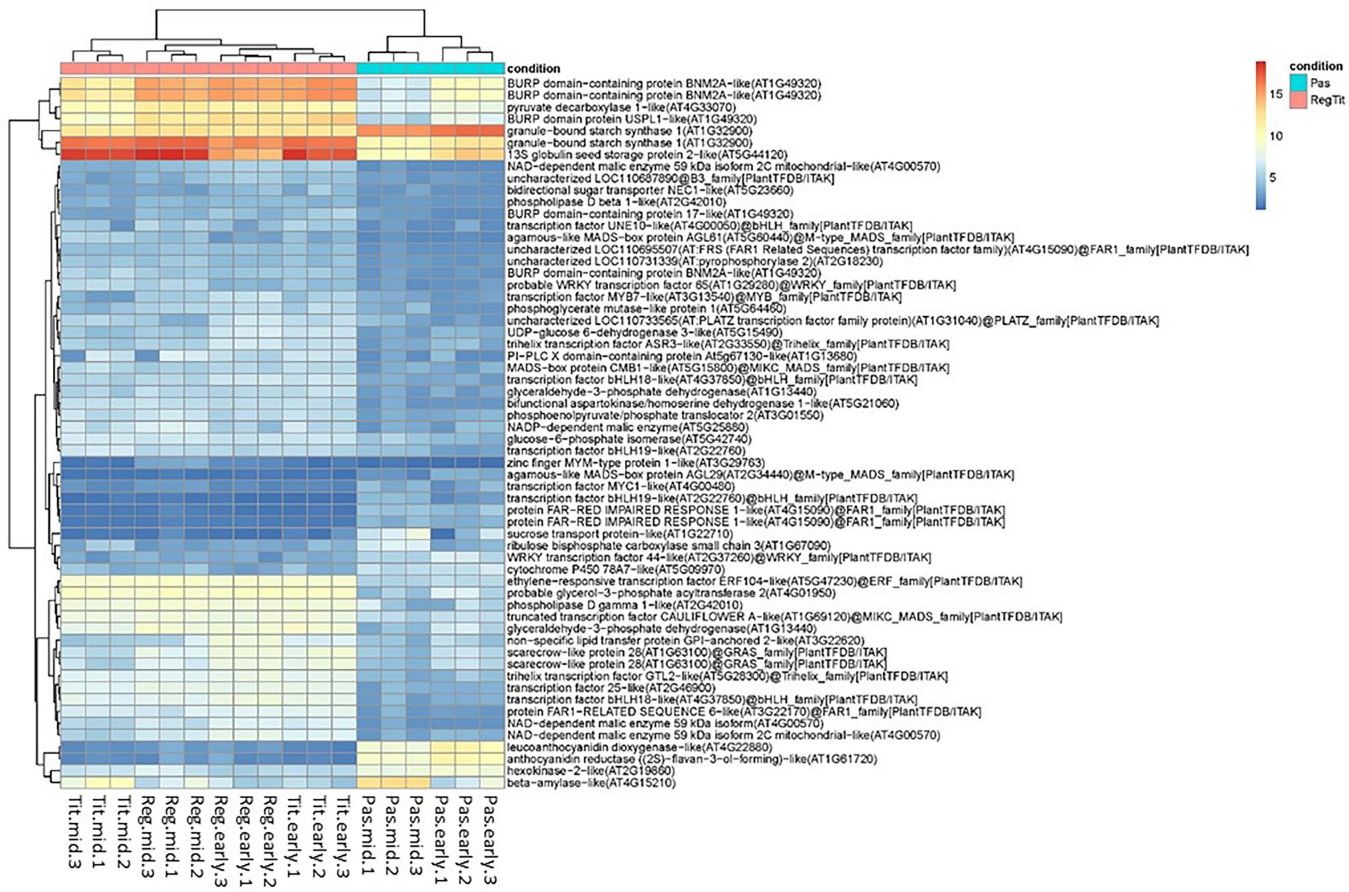
Heat map showing the expression levels of the 60 selected transcripts (rlog normalized scores) in quinoa seeds of potential importance for regulating seed quality in Pasankalla. These selected transcripts encode either functions involved in central carbon metabolism (sugar, starch, lipid, and protein metabolism) or transcription factors, and they all showed at least eight times higher/lower transcript expression (log2FC > 3.0, value of *p* ≤ 0.01) in Pasankalla (Pas, turquiose) as compared to in Regalona and Titicaca (RegTit, pink). Expression levels shown are normalized raw read counts (as from the mapping onto the reference genome) using regularized logarithm (rlog). Values are shown from triplicate biological samples from the early and mid-developmental stages (for corresponding dpa, see the text).

In an attempt to better understand the genetic differences between the quinoa genotypes that could explain the differences in seed quality, we investigated the encoded functions of the seed transcripts that were found to be differentially expressed between the genotypes. Supplementary Table S2 presents the list of the 3,072 DETs from the comparison of Pasankalla to Regalona and Titicaca and includes the expression values (estimated raw read counts) in the biological replicates, log2fold changes, and the functional annotations of the transcripts. From these DETs, we further filtered out the most highly DETs in Pasankalla as compared to in the other genotypes, i.e., the transcripts that exhibited at least eight times higher/lower transcript expression (by applying new stringent threshold of log2FC > 3.0). In the resulting list of 999 DETs (Supplementary Table S3), we manually identified all the transcripts that encoded either transcription factors (regulatory genes) or functions that had a high probability of having a large impact on carbon allocation between different seed storage products or tissues. These latter functions included the central carbon metabolism of starch, sugar, protein, and oil, and genes known to regulate embryo size. In total, 60 target transcripts were identified, for which the rlog normalized scores of the expression levels in seeds of the three quinoa genotypes are shown as a heat map in Figure 8 (and can also be found as a separate list in Supplementary Table S3).

### Differential Gene Expression in the Central Carbon Metabolism and Embryo Size Regulating Gene

Among the 60 target transcripts that were found to be highly differentially expressed in Pasankalla as compared to the other varieties, many were involved in the central carbon metabolism, and one was possibly involved in regulating embryo size. In the description of several of these target genes below, the names and gene identification numbers of their closest homologs in Arabidopsis are given in parentheses, as the genome of this model plant species is still one of the most thoroughly studied and annotated. For certain target transcripts, the seed tissue specificity of the gene expression of the corresponding homologs in Arabidopsis is also given from available data (Winter et al., 2007) to aid the interpretation of whether these target genes could be of importance for seed development and, therefore, seed quality.

One transcript that encodes a sucrose transporter (Chen et al., 2015) was down-regulated (SWEET12, AT5G23660, expressed in the suspensor at an early seed developmental stage), and a transcript for another sucrose transporter (Srivastava et al., 2008) was up-regulated (SUCROSE-PROTON SYMPORTER 2, AT1G22710, expressed in the endosperm at a later stage), in Pasankalla as compared to the other genotypes. Of the nine transcripts that encoded glycolytic functions, only the one for hexokinase (HEXOKINASE 2, AT2G19860, expressed in the micropylar endosperm at an early stage and in the chalazal seed coat at a later stage), which catalyzes the phosphorylation of glucose and fructose (Karve et al., 2008), was up-regulated in Pasankalla. The other glycolytic functions, that are downstream of sucrose, were all down-regulated (e.g., transcripts for glucose-6-phosphate isomerase, glycerol-aldehyde-3-phosphate dehydrogenase, phosphoglycerate mutase, malic enzyme, and pyruvate decarboxylase). Further, a transcript encoding a phosphoenolpyruvate translocator (Knappe et al., 2003) that allows for the transportation of three-carbon moieties across the chloroplast membrane (PHOSPHOENOLPYRUVATE/PHOSPHATE TRANSLOCATOR 2, AT3G01550, mainly expressed in the seed coat at a mid-stage of development) was also found to be down-regulated.

Interestingly, a transcript that encodes a subunit of the ribulose-bisphosphate carboxylase/oxygenase (RUBISCO SMALL CHAIN 1A, AT1G67090, expressed in the endosperm during all stages of seed development and in the embryo at the later stages), which plays a central role in carbon fixation in the Calvin cycle (Izumi et al., 2012), was up-regulated in Pasankalla as compared to the other genotypes. The starch metabolism transcripts also showed differential expression in Pasankalla, which potentially indicates turnover of starch, with the up-regulation of a transcript that encodes a granule bound starch synthase [GRANULE BOUND STARCH SYNTHASE 1 (GBSS1), AT1G32900, expressed in the seed coat] and of another that encodes a beta-amylase, which is responsible for starch degradation (BETA-AMYLASE, AT4G15210, similar expression pattern as that for GBSS1; Streb and Zeeman, 2012). It should be noted that a second transcript that encodes a granule bound starch synthase (which also has AT1G32900 as the closest Arabidopsis homolog) was down-regulated, which is opposite of the case of the first transcript mentioned. With regard to lipid metabolism, two transcripts that encode phospholipases (PHOSPHOLIPASE D BETA 1, AT2G42010, expressed in the chalazal endosperm at an early stage) and another transcript that encodes an acyltransferase involved in the synthesis of precursors for oil synthesis (GLYCEROL-3-PHOSPHATE SN-2-ACYLTRANSFERASE, AT4G01950; Li-Beisson et al., 2013) were all down-regulated in Pasankalla as compared to the other genotypes.

Several down-regulated transcripts that are involved in storage protein accumulation were found in Pasankalla as compared to the other genotypes. One encoded a seed storage protein that was annotated from the quinoa reference genome to a 13S globulin (XP_021752668.1) and had CRUCIFERIN as the closest Arabidopsis homolog (a 12S seed storage protein, AT5G44120, expressed in the embryo at a later stage). Another transcript encoded a protein that possibly play the role of a 2S albumin storage protein according to the annotation of the closest Arabidopsis homolog (AT3G22620, expressed in the seed coat at a later stage). Four other transcripts encoded proteins for which the closest homolog in Arabidopsis was the UNKNOWN SEED PROTEIN LIKE 1 (AT1G49320). This is a BURP domain containing protein, targeted to the protein storage vacuoles which has been shown to be important for proper seed development and protein storage (Van Son et al., 2009).

Finally, and interestingly, one up-regulated transcript in Pasankalla had the closest Arabidopsis homolog being CYP78A7 (AT5G09970) that encodes a cytochrome P450. The closely related CYP78A13 is encoded by the gene *GIANTEMBRYO* in rice *Oryza sativa* that is known to regulate embryo size (Nagasawa et al., 2013).

### Differential Gene Expression of Transcription Factors

Several of the remaining transcripts on the list of the selected candidate genes that showed high differential expression in Pasankalla as compared to the other genotypes were found to be encoding transcription factors, of which several encoded AGAMOUS-LIKE proteins. One example was a down-regulated transcript, for which the closest homolog in Arabidopsis was AGAMOUS-LIKE 62 (AT5G60440, expressed in the peripheral and chalazal endosperm), that suppresses the cellularization of the endosperm (Hehenberger et al., 2012; Figueiredo et al., 2015; Xu et al., 2016). Another example of a down-regulated transcript in Pasankalla was one that was homologous to AGAMOUS-LIKE 29 (AT2G34440, expressed in the peripheral and micropylar endosperm at an early developmental stage). An additional down-regulated transcript in Pasankalla encoded a protein that was homologous to AGAMOUS-LIKE 7/APETALA1 (AT1G69120, not highly expressed in seed tissues) and is known to be involved in the regulation of floral identity of meristems (Mandel et al., 1992; Weigel and Meyerowitz, 1993).

Several transcripts involved in the far red light response signaling were differentially expressed in Pasankalla as compared to the other genotypes. Three transcripts encoded proteins for which the closest Arabidopsis homolog was FAR-RED IMPAIRED RESPONSE 1 (AT4G15090), with two of the transcripts being up-regulated, while the third was down-regulated. An additional down-regulated transcript encoded a protein for which the closest Arabidopsis homolog was CHLOROPLAST DIVISION MUTANT 45 (AT3G22170), which is a part of the phytochrome A-dependent signaling network for responses to far-red light (Wang and Deng, 2003).

As mentioned previously, the seed coat of Pasankalla most probably contained more pigments than the other genotypes, which was observed through visual inspection of the seeds and seed flour (Figure 3). Interestingly, one up-regulated transcript in Pasankalla was found to encode a transcription factor for which the closest homolog in Arabidopsis was WRK44/DR STRANGELOVE/TRANSPARENT TESTA GLABRA 2 (AT2G37260), which is known to be involved in the synthesis of seed coat tannins and affect seed color (Johnson et al., 2002). Anthocyanins and flavonoids are examples of other phenolic compounds that are common in the pigments of different plant tissues. Two transcripts that encode functions involved in pigment synthesis, namely the BANYULS (AT1G61720), which is a reductase in Arabidopsis that is important in seed coat flavonoid synthesis (Albert et al., 1997), and the ANTHOCYANIDIN SYNTHASE (AT4G22880), which is involved in the synthesis of both seed and leaf pigments (Liu et al., 2013; Hong et al., 2017; Zheng et al., 2019), were up-regulated in Pasankalla. The closest homologous genes to these three transcripts in Arabidopsis are highly expressed in the seed coat.

Several other identified DETs in Pasankalla were encoding transcription factors for which the homologs in Arabidopsis are expressed in the seed coat. These were, for example, AGAMOUS-LIKE 2/SEPALLATA1 (AT5G15800), which is involved in flower and ovule development (Favaro et al., 2003), and MYB DOMAIN PROTEIN 5 (AT3G13540), which is a negative regulator of trichome branching (Song et al., 2009) and important for proper seed coat development (Gonzalez et al., 2009). The corresponding transcripts of these genes were both down-regulated in Pasankalla seeds. Another transcript that was found to be involved in the regulation of trichome development, but was up-regulated instead of down-regulated in Pasankalla, encoded a protein that was homologous to Arabidopsis MYC1 (AT4G00480, expressed in the embryo; Morohashi and Grotewold, 2009). Finally, in the target transcript list, there were also a few that encoded transcription factors for which the closest homologs in Arabidopsis do not have well-known functions and do not exhibit high expression in seed tissues. These belong to the basic helix–loop–helix (bHLH) DNA-binding superfamily (AT2G22760, AT4G37850); one encoded a SH4-RELATED 3 protein (AT2G33550), while another encoded PHYTOCHROME INTERACTING FACTOR 8 (AT4G00050). Further, one encoded ETHYLENE RESPONSIVE ELEMENT BINDING FACTOR 5 (AT5G47230). Lastly, one target transcript encoded an uncharacterized protein with no hit in the Arabidopsis genome.

To get a complementary global view of which processes were represented in the whole set of 3,568 transcripts identified to be differentially expressed in Pasankalla as compared to the other two genotypes, we made a GO term enrichment analysis. The result showed that the 3,568 DETs represented 48 different GO terms of which the majority (36) were in biological process, seven in cellular component, and five in molecular function (Supplementary Table S4). The 60 candidate genes discussed above (Figure 8) could be associated to 24 of the 48 GO terms listed (see GO terms marked yellow in Supplementary Table S4). Finally, we also made a pathway enrichment analysis based on this list of 3,568 DETs in Pasankalla and the KEGG pathway database, which showed that flavonoid biosynthesis pathway was highly enriched as compared to the other genotypes (Supplementary Figure S3).

### SNP Identification in Differentially Expressed Genes

In an attempt to identify genetic markers that could be important for quinoa seed quality, we analyzed the RNA sequencing data for the presence of SNPs/InDels (hereafter also called short variants) in the transcripts that were identified to be differentially expressed in the high-protein containing Pasankalla as compared to the other genotypes. This was carried out through a sequential filtering of SNPs/InDels, an overview of which is presented in Table 2. First, we identified all the available short variants by comparing the sequence of RNA reads to the reference genome. In general, the Pasankalla transcriptome showed a much higher number of short variants (341,911) as compared to Regalona (151,816) and Titicaca (126,847). After stringent quality filtering, approximately 40% of these short variants were included in the next step. The number of target SNPs/InDels was further reduced by filtering out those that were present within the 3,072 transcripts that were previously identified to be DETs in Pasankalla. It can be noted that Pasankalla presented a much higher number of SNPs/InDels located within the identified DETs (8,939 in the 3,072 DETs) as compared to Regalona (442 in the 330 DETs) and Titicaca (416 in the 332 DETs). The number of target SNPs/InDels located in the DETs of Pasankalla was finally reduced by identifying which of them were high-to-moderate impact variants, which refers to those that lead to amino acid exchanges, introduction or deletion of a start or stop codon, open reading frame shifts, and alternative splicing. Such variants are highly likely to change the function of the encoded protein. Our analysis revealed that 2,863 such short variants were found in the Pasankalla seed transcriptome (Supplementary Table S5), while only 115 were found in Regalona and 142 in Titicaca. The identification of a higher number of high-to-moderate impact SNPs/InDels in the DETs of Pasankalla as compared to the other genotypes indicated that Pasankalla is most probably genetically more distant to the reference genome and to the two other genotypes. Genetic distance between highland and coastal quinoa has previously been shown by Jarvis et al. (2017).

**Table 2:** Number of sequence variations (SNPs/InDels) in quinoa seed transcriptomes as compared to the quinoa reference genome.

Explanation of identified SNPs/InDels	Pasankalla	Regalona	Titicaca
Total SNPs/InDels in transcripts after alignment to reference genome	341,911	151,816	126,847
Subset of SNPs/InDels after quality filters	148,863	67,446	45,456
Subset of SNPs/InDels after quality filters that are present within significantly DETs	8,939	442	416
Total no of High and Moderate impact variants	2,863	115	142
*Splice acceptor variant*	47	2	6
*Splice donor variant*	47	5	4
*Stop gained*	68	3	4
*Frameshift variant*	219	10	11
*Stop lost*	14	1	1
*Start lost*	15	0	0
*Missense variant*	2,473	97	119

*Numbers given are: total number of SNPs/InDels in the transcriptomes of the three genotypes (when individually compared to the reference genome) and number of these SNPs/InDels left after quality filtering [QUAL (The Phred-scaled probability that a REF/ALT polymorphism exists at this site given sequencing data) ≥30; Read Depth ≥10; Genotype Quality ≥20]. Further the number of the quality filtered SNPs/InDels that were found in the significantly (p < 0.01 and Fold Change >1) DETs were extracted, and finally the numbers of these identified SNPs/InDels that was yielding high and moderate impact amino acid changes. For example, the seed transcriptome of Pasankalla showed 341,911 SNPs/InDels as identified from comparison to the quinoa reference genome, of which 148,863 SNPs/InDels were left after quality filtering. Of these 148,863 SNPs/InDels, 8,939 were found in the 3,072 significant DETs identified in Pasankalla as compared to Titicaca and Regalona (see* Table 1*). Of these 8,939 SNPs/InDels in DETs, 2,863 SNPs/InDels were identified as being of high to moderately impacting variants giving rise to splice acceptor/donor variants, introducing or deleting stop/start codons, frameshift and missense variants.*

The list of the 2,863 high-to-moderate impact short variants identified in Pasankalla should be the most interesting for further evaluation and development into genetic markers for protein content that could also potentially be associated to embryo size. Therefore, we took a closer look at the genes in which these 2,863 SNPs/InDels were located, i.e., whether any of these were found in the target list of 60 transcripts that were identified as being differentially expressed in Pasankalla. In fact, SNPs/InDels were present in 19 of the candidate transcripts (Table 3). These short variants are described below, and the function of the genes in which they were found have already been described above.

**Table 3:** List of moderate to high impact SNPs/InDels found in Pasankalla seed transcripts as compared to the reference genome in 19 of the 60 candidate transcripts (listed in Supplementary Table S5).

#CHROM	POS	REF	ALT	QUAL	Genotype	Predicted effects	NG-14833_Pas	Gene annotation	Closest *Arabidopsis* homolog
NW_018742204.1	12313015	C	T	2062.77	1/1	Missense_variant	rna498	Protein FAR-RED IMPAIRED RESPONSE 1-like	AT4G15090
NW_018742204.1	12313702	A	G	4467.77	1/1	Stop_lost&splice_region_variant	rna498	Protein FAR-RED IMPAIRED RESPONSE 1-like	AT4G15090
NW_018742204.1	19431791	G	A	3625.77	1/1	Stop_gained	rna1048	Truncated transcription factor CAULIFLOWER A-like	AT1G69120
NW_018742205.1	1222228	A	T	1548.77	0/1	Splice_acceptor_variant&intron_variant	rna1790	Granule-bound starch synthase 1	AT1G32900
NW_018742205.1	1223861	A	G	154458.77	1/1	Missense_variant	rna1790	Granule-bound starch synthase 1	AT1G32900
NW_018742205.1	1224425	G	C	101104.77	1/1	Missense_variant	rna1790	Granule-bound starch synthase 1	AT1G32900
NW_018742205.1	1225082	A	G	29044.77	1/1	Missense_variant	rna1790	Granule-bound starch synthase 1	AT1G32900
NW_018742418.1	4213817	C	A	1850.77	1/1	Splice_donor_variant&intron_variant	rna6695	Glucose-6-phosphate isomerase	AT5G42740
NW_018742418.1	4213846	A	G	2701.77	1/1	Missense_variant	rna6695	Glucose-6-phosphate isomerase	AT5G42740
NW_018742418.1	4214066	A	G	2573.77	1/1	Missense_variant	rna6695	Glucose-6-phosphate isomerase	AT5G42740
NW_018742418.1	6800112	C	T	382.77	0/1	Missense_variant	rna6969	Trihelix transcription factor ASR3-like	AT2G33550
NW_018742418.1	6800393	G	T	992.77	1/1	Missense_variant	rna6969	Trihelix transcription factor ASR3-like	AT2G33550
NW_018742484.1	3420361	G	T	3626.77	1/1	Splice_acceptor_variant&intron_variant	rna8863	Leucoanthocyanidin dioxygenase-like	AT4G22880
NW_018743014.1	1550482	C	A	346.77	0/1	Splice_acceptor_variant&intron_variant	rna20892	Hexokinase-2-like	AT2G19860
NW_018743033.1	1533172	A	G	3475.77	0/1	Missense_variant	rna21577	Pyruvate decarboxylase 1-like	AT4G33070
NW_018743033.1	1534233	T	C	4599.77	0/1	Missense_variant	rna21577	Pyruvate decarboxylase 1-like	AT4G33070
NW_018743033.1	1535169	G	C	7051.77	0/1	Missense_variant	rna21577	Pyruvate decarboxylase 1-like	AT4G33070
NW_018743066.1	1868796	A	G	717.77	1/1	Missense_variant	rna22494	Uncharacterized LOC110731339(AT:pyrophosphorylase 2)	AT2G18230
NW_018743175.1	1811370	C	G	109.77	0/1	Missense_variant	rna23725	Ethylene-responsive transcription factor ERF104-like	AT5G47230
NW_018743175.1	1811380	A	T	112.77	0/1	Missense_variant	rna23725	Ethylene-responsive transcription factor ERF104-like	AT5G47230
NW_018743175.1	1811963	A	C	3684.77	1/1	Missense_variant	rna23725	Ethylene-responsive transcription factor ERF104-like	AT5G47230
NW_018743397.1	696295	G	C	27773.77	0/1	Missense_variant	rna31317	Granule-bound starch synthase 1	AT1G32900
NW_018744290.1	1355398	A	T	2531.77	0/1	Missense_variant	rna48879	BURP Domain-containing protein BNM2A-like	AT1G49320
NW_018744290.1	1355566	G	A	12661.77	0/1	Missense_variant	rna48879	BURP Domain-containing protein BNM2A-like	AT1G49320
NW_018744489.1	33930	T	TATCA	49.73	0/1	Frameshift_variant&stop_gained	rna51742	BURP Domain-containing protein BNM2A-like	AT1G49320
NW_018744878.1	3241449	T	C	2645.77	1/1	Missense_variant	rna60851	Protein FAR-RED IMPAIRED RESPONSE 1-like	AT4G15090
NW_018744878.1	3241962	G	C	1718.77	1/1	Missense_variant	rna60851	Protein FAR-RED IMPAIRED RESPONSE 1-like	AT4G15090
NW_018744878.1	3241977	CCGGCATATAACATGGCACCA	C	1003.73	1/1	Frameshift_variant	rna60851	Protein FAR-RED IMPAIRED RESPONSE 1-like	AT4G15090
NW_018744878.1	3242167	C	A	1792.77	1/1	Missense_variant	rna60851	Protein FAR-RED IMPAIRED RESPONSE 1-like	AT4G15090
NW_018744955.1	1064185	A	G	17049.77	1/1	Missense_variant	rna61870	Transcription factor bHLH19-lik	AT2G22760
NW_018744955.1	1064321	T	C	14898.77	1/1	Missense_variant	rna61870	Transcription factor bHLH19-like	AT2G22760
NW_018745003.1	393540	A	T	472.77	0/1	Missense_variant	rna63362	NADP-dependent malic enzyme	AT5G25880
NW_018745003.1	393541	A	T	472.77	0/1	Missense_variant	rna63362	NADP-dependent malic enzyme	AT5G25880
NW_018745323.1	1054649	C	T	2359.77	0/1	Missense_variant	rna68911	Glyceraldehyde-3-phosphate dehydrogenase	AT1G13440
NW_018745454.1	1606358	C	CACCACCGG	1039.73	1/1	Frameshift_variant	rna70849	Phospholipase D gamma 1-like	AT2G42010
NW_018745454.1	1606396	C	G	919.77	1/1	Missense_variant	rna70849	Phospholipase D gamma 1-like	AT2G42010
NW_018745454.1	1606841	G	A	1495.77	1/1	Missense_variant	rna70849	Phospholipase D gamma 1-like	AT2G42010
NW_018745454.1	1607164	C	CA	1286.73	0/1	Frameshift_variant	rna70849	Phospholipase D gamma 1-like	AT2G42010
NW_018745454.1	1607221	A	G	2132.78	1/1	Missense_variant	rna70849	Phospholipase D gamma 1-like	AT2G42010
NW_018745454.1	1607446	G	A	2668.77	1/1	Missense_variant	rna70849	Phospholipase D gamma 1-like	AT2G42010
NW_018745454.1	1607710	A	G	1366.77	1/1	Missense_variant	rna70849	Phospholipase D gamma 1-like	AT2G42010
NW_018745454.1	1610173	T	A	2160.77	1/1	Splice_donor_variant&intron_variant	rna70849	Phospholipase D gamma 1-like	AT2G42010
NW_018745454.1	1613459	C	G	4019.77	0/1	Missense_variant	rna70849	Phospholipase D gamma 1-like	AT2G42010
NW_018745684.1	2622955	C	T	11608.77	0/1	Missense_variant	rna74938	13S Globulin seed storage protein 2-like	AT5G44120
NW_018745684.1	2624227	C	G	6071.73	0/1	Missense_variant	rna74938	13S Globulin seed storage protein 2-like	AT5G44120

#CHROM; assembly scaffold numbers, POS; base position in scaffolds, REF; DNA base in reference genome, ALT; DNA base in Pasankalla seed transcript, QUAL; quality score, Genotype; 1/1 means that Pasankalla is homozygote for the identified SNP/InDel and 0/1 means Pasankalla is heterozygote for the SNP/InDel, Predicted effects; type of SNP/InDel, NG-14833_Pas; Pasankalla transcript number, Gene annotation; functional annotation from the quinoa genome, Closest *Arabidopsis* homolog; gene identification number of closest homolog in *Arabidopsis thaliana*.

Several SNPs/InDels were located in the genes that encoded functions in the central carbon metabolism. Two missense SNPs (yielding amino acid exchanges) were found in the transcript that encoded a 13S globulin seed storage protein, and, additionally, three variants (two missense SNPs and one insertion of ATCA that causes a frameshift) were found in the two transcripts for which the closest Arabidopsis homolog encoded UNKNOWN SEED PROTEIN LIKE 1. The transcript that encodes a granule-bound starch synthase and was highly up-regulated in Pasankalla contained four SNPs (three missense and one that causes alternative splicing), and the transcript that encoded the same enzymatic function but was highly down-regulated contained one missense SNP. Further, missense SNPs were also identified in the transcripts that encoded the glycolytic functions (glucose-6-phosphate isomerase, glyceraldehyde-3-phosphate dehydrogenase, pyrophosphorylase, malic enzyme, and pyruvate decarboxylase), and SNPs that cause alternative splicing were found in the transcripts for glucose-6-phosphate isomerase and hexokinase. A SNP that leads to alternative splicing was identified in the transcript that encoded a synthase involved in proanthocyanin biosynthesis. Finally, the remaining nine SNPs/InDels on the list that are associated to genes involved in the central carbon metabolism were all found in one transcript that encodes a phospholipase D beta 1-like protein. Six of those were missense SNPs, two were insertions (of A and of ACCACCGG) that both cause frameshifts, and the last one was a SNP that causes alternative splicing.

Six transcription factors were represented in the list of the 19 target transcripts that carried SNPs/InDels. One of those was the CAULIFLOWER A-like, the closest homolog in Arabidopsis for which is AGAMOUS-LIKE 7, in which one SNP was identified that introduced a stop codon. Another transcription factor was involved in signaling to light, namely FAR-RED IMPAIRED RESPONSE 1, in which two SNPs were found in one transcript (one missense SNP, and the other SNP causing the loss of a stop codon), and three missense SNPs were found in another transcript, in which a deletion of 20 bases was also observed, which caused a frameshift. A third transcription factor on the list was one for which the closest homolog in Arabidopsis was ETHYLENE RESPONSIVE ELEMENT BINDING FACTOR 5, in which three missense SNP variants were identified. Finally, two missense SNPs each were associated with the transcripts that encode a trihelix transcription factor (for which the closest homolog in Arabidopsis was SH4-RELATED3) and a bHLH DNA-binding superfamily protein.

## Discussion

This study aimed to provide a better understanding of the genetic regulation of quinoa seed quality, in general, and protein content, in particular, by studying three quinoa genotypes of diverse origin: the highland landrace Pasankalla and the cultivars Regalona and Titicaca that originate from coastal types. These genotypes, which were previously identified to have different protein seed content at maturity (Gargiulo et al., 2019), were here characterized through comparative analyses of nutrient (starch, protein, and oil) accumulation and gene expression profiles during seed development under controlled growth conditions.

### Genotype Differences in Seed Carbon Allocation in Quinoa

Analyses of seed metabolites in different ecotypes of quinoa have previously revealed significant differences between coastal and highland ecotypes (Pinto-Irish et al., 2020). In our study, we found that the three quinoa genotypes had different seed protein content at maturity, as previously described (Gargiulo et al., 2019), and our analyses of nutrient accumulation in developing seeds showed that they had different levels of the other major storage compounds starch and oil as well. The final levels of the major storage nutrients in the mature seed is the result from an integration of the biochemical programs during seed development. In some plant species, such as in Arabidopsis, starch is transiently accumulating during seed development and then mobilized to feed oil biosynthesis with carbon, which results in high oil levels but very low levels of starch, in the mature seed (Baud et al., 2002). From our data on developing quinoa seeds no such transient accumulation of any of the major storage compounds was observed. The genotype Pasankalla showed the highest seed protein content (20%), the highest oil content (3.8%), and the lowest starch content (46%) at maturity. This result could indicate that there is a negative correlation of both protein and oil content to starch content in quinoa, as known to be the case for example in oat (Peterson and Wood, 1997), but screening of a large number of quinoa genotypes would be necessary to confirm such hypothesis. It should be noted that, although the oil content in Pasankalla was statistically higher than that in the other genotypes only at an early stage of seed development, the observed increased proportion of oleic acid (18:1) in the seed oil from Pasankalla suggests that this genotype actually has increased oil synthesis. This is because an increased oleic acid proportion in seed oil has previously been associated with an increased capacity for oil synthesis in other plant species such as oat (Banas et al., 2007). The amino acid composition of flour from mature seeds was comparable to previously reported data (Dakhili et al., 2019) and only showed small genotype differences.

To define efficient breeding strategies with regard to seed quality, it is important to know the specific seed tissues in which the different storage compounds of interest are deposited, as this can differ between crops and even different varieties of the same crop (Baud and Lepiniec, 2010). The majority of proteins and oil that accumulate in quinoa seeds are deposited in the embryo tissue, while starch accumulates in the perisperm as opposed to the endosperm, which is the case for cereals (Burrieza et al., 2014). Interestingly, in a previous study that used X-ray microtomography to examine mature seeds from the same quinoa genotypes under the same growth conditions, the increased protein content was associated to a larger embryo proportion (Gargiulo et al., 2019). Therefore, it was not surprising that the genotype that showed the highest protein content also had the highest oil content, because these compounds co-accumulate in the embryo tissue. This shows that these quinoa genotypes vary in terms of seed carbon allocation to the different storage nutrients and that this, at least partially, can be due to a variation in the distribution of carbon between the different seed tissues. From a plant breeding perspective, an increased protein content in quinoa could probably be achieved by aiming to obtain an enlarged embryo. Attempting to increase the proportion of specific seed tissues to achieve a desired seed quality is an approach that has been previously used for several cereal crops. For example, enlarged embryos in maize kernels resulted in increased oil content by recurrent selection (Alexander and Seif, 1963), and mutants with giant embryo phenotypes have been isolated to increase the nutritional value of rice (Nagasawa et al., 2013; Lee et al., 2019). Due to the genotype differences in seed quality observed in our study, these three quinoa genotypes can be used as a model system for identifying genetic factors that regulate embryo size and, therefore, protein content in quinoa.

We performed our study in a controlled growth environment with a day-length of 12 h in which all three genotypes flowered and developed seeds, which allowed for a comparison under the same growth conditions. However, it should be noted that genotype differences in day-length sensitivity can lead to phenological differences that could potentially have an effect on seed composition. Pasankalla is a highland type and the cultivars Regalona and Titicaca both originate from coastal regions in Chile. All three analysed genotypes were flowering at approximately the same time after germination, but the seed development in Pasankalla was then delayed about 13 days, as compared to the other genotypes. The time points for sampling of the mid, early, and late seed developmental stages in Pasankalla were therefore adjusted by visual inspection of seeds, and seed dry matter content confirmed that the sampled stages were comparable for all genotypes. These three stages are all found in “phase 2” of quinoa seed development which is during cell expansion and storage accumulation (López-Fernández and Maldonado, 2013). Prolonged seed development caused by differences in photoperiod sensitivity has previously been reported for quinoa where the seeds of a genotype adapted to short days developed slower or never reached maturity under long day conditions (Christiansen et al., 2010; Adolf et al., 2012). However, these studies did not consider seed quality aspects, and future research needs to address the effect of such prolonged seed development window, as well as that of other environmental factors, on quinoa seed quality.

### Comparative Transcriptomics of Quinoa Seeds to Identify Candidate Genes for Marker Development

RNA sequencing was performed for developing quinoa seeds to obtain a better understanding of the genes that were differentially expressed in Pasankalla, which had the highest protein content as compared to the other genotypes. For this, we used the recently sequenced genome of quinoa as a reference (Jarvis et al., 2017). The first approach that employed genotype pairwise comparisons of the transcriptomes revealed a much higher number of DETs when comparing Pasankalla to the two other genotypes in contrast to when comparing Regalona to Titicaca (Figure 6A). Therefore, a second approach was used in which the seed transcriptome from each genotype was compared to the two others together to filter out the most interesting target genes in Pasankalla. This second approach clearly showed that Pasankalla could be distinguished from the other genotypes, as a much higher number (3,568) of DETs were found when contrasting Pasankalla with Regalona and Titicaca, as compared to the 361 and 374 DETs that were identified for the other comparisons (Figure 6B). Through a sequential filtering of these 3,568 DETs and, finally, a manual selection based on annotation information, we could identify 60 candidate transcripts that were involved in the central carbon metabolism or encoding transcription factors that were highly differentially expressed (at least eight times higher or lower) in Pasankalla as compared to the other genotypes. We used the extensively studied and annotated model species Arabidopsis as a major reference when interpreting our seed transcriptome data, but it should be noted that those annotations were in most cases in accordance with those available from the quinoa genome.

Altogether, our data revealed large varietal differences in the expression levels of quinoa seed transcripts in Pasankalla that are encoding transcription factors and functions in the sugar, starch, lipid and protein biosynthesis pathways, for which the closest Arabidopsis homologs are highly expressed in different and specific seed tissues. These transcripts can be expected to have significant effects on seed quality in general and, therefore, should be interesting gene candidates for developing genetic markers within, or upstream of, the coding region. It can be noted that the genetic differences found could possibly be related to the evolution (Jarvis et al., 2017) and state of selection of the studied plant materials (one landrace and two cultivars) or the origins of the same (one from highlands and two from coastal regions).

### Candidate Genes in the Central Carbon Metabolism and in Regulation of Embryo Size

A developing seed is a sink tissue that is dependent on the import of carbon in the form of sucrose from other photosynthetically active parts (source tissues) of the plant. The transport and import of sugar into the developing seed endosperm and embryo tissues from the surrounding maternally originating seed coat is therefore of substantial importance for proper seed filling and, ultimately, yield (Braun et al., 2014; Lafon-Placette and Köhler, 2014). In our target gene list, we found several highly DETs in Pasankalla, as compared to the other genotypes that were encoding functions in sugar transport. The homologs closest to these in Arabidopsis were SUCROSE-PROTON SYMPORTER 2 and SWEET12, both of which are highly expressed in different specific seed tissues and at different developmental stages (Winter et al., 2007). SWEET proteins have previously been shown to be of high importance for sugar delivery into and the proper filling of developing Arabidopsis seeds (Chen et al., 2015). The large differential expressions that we observed between the genotypes in the corresponding quinoa genes could therefore potentially affect seed quality through a changed carbon allocation between the specific seed tissues.

Cereal endosperm is a major source of storage starch (Huang et al., 2021) and starch is also the major storage compound in quinoa seeds where it is mostly stored in the perisperm. Large changes in the expression of genes that govern starch synthesis in seeds can therefore be expected to have an impact on seed quality. In fact, the transcripts that encode a granule-bound starch synthase as well as a starch degrading enzyme (beta-amylase) were highly up-regulated in Pasankalla, which could indicate starch turnover. At the same time, there was another transcript that also encodes a granule-bound starch synthase but was found to be highly down-regulated instead. It could be speculated that these two different transcripts represent two different starch synthases in quinoa that are of importance for different seed tissues (i.e., for transitory or storage starch), such as those that have been reported in monocots (Jeon et al., 2010). Pasankalla had the lowest starch content, and it can be speculated that this is due to an increased competition for carbon which, in this genotype, is channeled into protein and oil to a higher extent. Related to carbon metabolism in general, it was also interesting to note that a transcript that encodes RubisCO was down-regulated in Pasankalla. This protein plays a central role in the Calvin cycle during photosynthetic reactions and has also been found to increase carbon efficiency in seeds that are green during development by rescuing carbon that is “wasted” from the energetically expensive fatty acid synthesis (Schwender et al., 2004).

The protein content of seeds is highly diverse among different plant species. Among our common crops today, soybean has the highest protein content of around 45% by seed dw (Patil et al., 2018). In soybean, the protein is stored in the embryo (cotyledons), which comprises the majority of the mature seed. In quinoa, the majority of the protein is stored in the embryo as well, but the embryo in this species usually comprises only around 25% of the seed volume (Gargiulo et al., 2019). In fact, embryo enrichment is an approach used when attempting to increase the protein content of flour obtained from the dry fractionation of quinoa seeds (Avila Ruiz et al., 2016). Breeding to achieve increased protein content in quinoa could, therefore, include one or several of the following objectives: enlarged embryo, increased protein content of the embryo, and increased protein content of the perisperm. The signal exchange between the embryo and endosperm during seed development can control the size of these tissues, which, in turn, affects seed quality (Nagasawa et al., 2013; Lafon-Placette and Köhler, 2014). Interestingly, in our study, we found that a transcript that is homologous to the Arabidopsis *CYP78A7*, which is closely related to the rice *CYP78A13* that regulates embryo size, was down-regulated in Pasankalla. In rice, the CYP78A13 is a cytochrome P450 protein that is encoded by the *GIANTEMBRYO* and has been shown to be expressed at the interphase of the embryo and endosperm, controlling cell size in the embryo and cell death in the endosperm and, thereby, regulating the size balance between these tissues (Nagasawa et al., 2013). The homologous transcript in quinoa is therefore highly interesting as a candidate gene for marker development to increase the protein content of quinoa.

Further, concerning seed protein content, our candidate gene list contained a down-regulated transcript in Pasankalla that was annotated to encode a 13S globulin storage protein. The major seed storage proteins in quinoa are usually considered to be the 11S globulins (chenopodin) and 2S albumins (Brinegar and Goundan, 1993; Balzotti et al., 2008; Thanapornpoonpong et al., 2008; Janssen et al., 2017; Dakhili et al., 2019) even though a 13S globulin (XP_021752668.1) was recently identified (Capraro et al., 2020). The observed downregulated transcript that encodes a 13S globulin storage protein in Pasankalla could possibly indicate that the higher seed protein content of this genotype should be attributed to other storage proteins. Further characterization of the protein fractions from the studied genotypes will be needed to determine this, but it can be noted that varietal differences in the seed globulins in quinoa were previously indicated in a study of 148 genotypes (Fairbanks et al., 1990). Other interesting transcripts were those that encode functions for which the closest homolog in Arabidopsis was UNKNOWN SEED PROTEIN LIKE 1 (AT1G49320). This is a protein that targets the protein storage vacuoles, which have been shown to be important for proper seed development and protein storage (Van Son et al., 2009). It is likely that such transcript differences between genotypes can affect the seed protein content.

Oil is another important storage compound in seeds of our common crops and the regulation of its synthesis and accumulation in plant seeds is complex involving many organelles, enzymes and lipid intermediates (Bates et al., 2013; Li-Beisson et al., 2013). Two transcripts that encode phospholipases that are involved in lipid metabolism (Li-Beisson et al., 2013), and in modulating diverse plant stress responses (Schnurbusch et al., 2019), were down-regulated in Pasankalla, and another down-regulated transcript encodes an acyltransferase that is involved in oil synthesis. These could potentially be factors that partly explain the higher oil content indicated in seeds of Pasankalla as compared to the other genotypes, although difficult to explain how. Intuitively one would expect that down-regulation of an acyltransferase involved in storage lipid biosynthesis could lead to a decrease in oil. However, due to the complexity of many biochemical pathways, such as for oil biosynthesis, the result of a changed biochemical step for the final levels of oil are not obvious. Further, some of the candidate genes identified in the comparative transcriptome study were annotated to have functions in glycolysis. It has previously been shown that, when changing the metabolic status in leaf tissues through the transient expression of a transcription factor (WRINKLED1) known to induce oil accumulation, transcripts encoding glycolytic functions were among the most affected, resulting in carbon being pushed through glycolysis to feed the increased fatty acid production (Grimberg et al., 2015). Thus, large changes in the gene expression patterns associated with glycolysis can probably also affect the allocation of carbon between different storage compounds during the development of seeds. However, there is a complex interplay between different transcription factors involved in seed developmental processes that can also be of importance for metabolic switches directed to oil synthesis (Fatihi et al., 2016; Rajavel et al., 2021).

### Candidate Genes That Encode Transcription Factors

All the aforementioned candidate genes are encoding proteins that serve enzymatic functions in specific biosynthetic pathways. However, genes that encode transcription factors are also highly interesting as candidate genes for marker development, since such proteins regulate the expression of whole sets or even “cascades” of genes, which can induce a strong effect on the phenotype (Kaufmann et al., 2010). Among the 60 candidate genes considered in this study, as many as 26 were encoding transcription factors. Several of these transcripts encoded proteins whose closest homologs in Arabidopsis were AGAMOUS-LIKE, which are known to be involved in seed developmental processes, such as ovule development and cellularization of the endosperm (Favaro et al., 2003; Hehenberger et al., 2012; Xu et al., 2016), and are therefore obviously interesting as candidate markers for seed quality.

Other transcripts were encoding regulatory proteins that are involved in the signaling network around the far-red light responses. Although our transcriptomes were based on developing seed tissues, which are usually not thought of as being the most important for a plant to integrate light signals, seeds are often green during development. The photosynthetic activity involved in developing green seed tissues has been suggested to contribute to an increased oxygen and energy status that can improve the carbon economy during the storage compound accumulation phase (Ruuska et al., 2004; Borisjuk and Rolletschek, 2009). It can be speculated that the gene expression differences found with regard to light signalling could be associated with different day-length sensitivities of the studied genotypes.

Our visual inspection of seed flour at a mid-developmental stage indicated that Pasankalla had more pigments than the other genotypes (Figure 3). Pasankalla was also previously shown to have a thinner seed coat and lower saponin content (Gargiulo et al., 2019) which is in agreement with a study that showed a positive correlation between those two traits in quinoa (Jarvis et al., 2017). Our study identified several highly differential expressed transcripts in Pasankalla that encoded functions that are of importance for the seed coat. One of these was the synthesis of seed coat tannins that affect seed color, WRK44/DR STRANGELOVE/TRANSPARENT GLABRA (Johnson et al., 2002) and two other transcripts were related to anthocyanin and flavonoid pigments. Further, another transcript associated with the seed coat, MYB DOMAIN PROTEIN 5, which was shown to be important for proper seed coat development (Gonzalez et al., 2009), was down-regulated in Pasankalla. This transcriptome data indicated that Pasankalla differs significantly concerning seed coat metabolism as compared to Regalona and Titicaca, which could affect the overall seed quality. In fact, our metabolite analyses showed that the total phenolic and total flavonoid levels among the studied genotypes were the highest in Pasankalla. Phenolic compounds, such as tannins, anthocyanins, and flavonoids, are secondary plant metabolites known to not only be involved in the pigmentation and protection of plant tissues but also to give antioxidant activity (Kähkönen et al., 1999; Kähkönen and Heinonen, 2003; Wojdyło et al., 2007; Repo-Carrasco-Valencia et al., 2010), which was found to be highest in Pasankalla in our analysis, as expected from the higher levels of phenolic and flavonoid compounds observed in this genotype. Flavonoid biosynthesis was also identified as the most activated pathway in Pasankalla as compared to the other genotypes, based on our KEGG pathway enrichment analysis. In a recent study targeting the flavonoid biosynthesis in quinoa, large differences in metabolites and transcriptomes were found between genotypes having seeds with diverse colors (Liu et al., 2022).

### SNPs/InDels in the Identified Candidate Transcripts Genes in Quinoa Seeds

Mutations in genes that encode transcription factors have been found to be common during the domestication process in many of our crops, which has affected fundamental traits that are important for agriculture development, such as plant and inflorescence architecture, flowering time, seed size, and seed shattering (Doebley et al., 2006). In our study, we identified SNPs/InDels that, in comparison to the reference genome, were unique to Pasankalla and located in the above presented candidate transcripts. In 19 out of the 60 candidate transcripts considered, we could identify short variants in Pasankalla that yield amino acid exchanges (missense SNPs), cause the introduction or deletion of a stop codon, and lead to alternative splicing. Such gene polymorphisms have a high possibility of significantly affecting (positively or negatively) the enzyme activity of the encoded protein and, by extension, the metabolic pathway or regulatory function in which it is involved which could in turn affect the plant phenotype such as seed quality.

Six of the candidate transcripts in which we found SNPs/InDels in Pasankalla were encoding transcription factors. The closest homologs in Arabidopsis to these genes were AGAMOUS-LIKE 7, FAR-RED IMPAIRED RESPONSE 1, ETHYLENE RESPONSIVE ELEMENT BINDING FACTOR 5, SH4-RELATED3, and a BHLH DNA-binding protein. As discussed above, several of these regulatory proteins have functions that are important for seed development, and the identification of SNPs in these genes that are unique to Pasankalla is therefore of great interest for further evaluation as markers.

The remaining 13 candidate transcripts on the list, in which short variants were found in Pasankalla, encoded functions in the central carbon metabolism. For example, several SNPs were identified in the transcripts that encode GRANULE-BOUND STARCH SYNTHASE, which plays a major role in the synthesis of starch, which is the dominant storage compound in quinoa seeds. It is noteworthy that genes that encode starch synthase are found in the list of genes in which mutations were proven to cause an important change in the domestication process of rice (Doebley et al., 2006). Other interesting examples of genes in our candidate list in which SNPs/InDels could possibly affect the seed protein phenotype of quinoa were the quinoa 13S globulin seed storage protein, and the BURP domain containing protein involved in the protein storage vacuole that is of importance for proper seed development. Additionally, SNPs/InDels were found in several of the candidate genes that encode glycolytic functions that are of major importance for allocating carbon in different suitable forms to feed the synthesis of seed storage compounds. Finally, and interestingly, as many as nine SNPs/InDels were found in one single gene that encodes a phospholipase D which is involved in phospholipid biosynthesis (Li-Beisson et al., 2013). It has been suggested that phospholipase D is involved in the flux of fatty acids into triacylglycerols through the formation of phosphatidic acid (Bates et al., 2020; Deepika and Singh, 2022), and the expression of a soybean phospholipase D in Arabidopsis enhanced the seed oil content (Bai et al., 2020). It could be speculated that the SNPs/InDels in the gene encoding a phospholipase D could possibly be associated with the increased oil content in Pasankalla as compared to the other genotypes. However, phospholipases are also involved in diverse signalling roles during plant abiotic and biotic stress responses (Schnurbusch et al., 2019; Deepika and Singh, 2022), and more detailed studies are therefore needed to confirm any functional association of phospholipase D to oil content in quinoa.

## Conclusion

Quinoa, as a crop, holds promise with regard to increased cultivation, especially in marginal lands in many parts of the world. Increased cultivation of this crop can contribute to a more diversified agriculture and can support the protein shift toward a more sustainable food production.

The identification of quinoa genotypes with contrasting seed quality can contribute a model system for the identification of precise breeding targets to improve the seed quality of quinoa. Altogether, our data from three quinoa genotypes identified highly up- or down-regulated transcripts in a genotype with enlarged embryo and increased protein content that encoded transcription factors or functions in seed metabolic pathways. The corresponding genes can be highly interesting candidates for the development of genetic markers for seed quality in quinoa. However, the effect of the candidate genes on the phenotypic differences observed between the investigated genotypes first needs to be validated. This could be performed by using targeted mutagenesis of candidate genes with CRISPR/Cas9-technology and/or by overexpressing the genes in quinoa, followed by characterization of seed quality. However, such molecular tools are still not available for quinoa (López-Marqués et al., 2020). The functional validation of candidate genes using such tools, or by using available mutants, could therefore be done in plant models such as Arabidopsis.

In total, we identified SNPs/InDels in 19 of the 60 candidate transcripts identified from comparative transcriptome analyses of the three quinoa genotypes with diverse seed quality. The SNPs/InDels we found were present in transcripts that encode both transcription factors and proteins involved in the central carbon metabolism and should have a high possibility of affecting quinoa seed quality. After having confirmed the importance of the suggested candidate genes for seed quality in quinoa using molecular tools in model organisms as described above, kompetitive allele specific PCR (KASP) markers for the detection of these short variants can be developed. These markers should, finally, be evaluated in a larger panel of quinoa genotypes to further elucidate their use as markers in quinoa breeding programs.

## Data Availability Statement

The original contributions presented in the study are publicly available. This data can be found at: National Center for Biotechnology Information (NCBI) BioProject database under accession number PRJNA596948.

## Author Contributions

AC, ÅG, RR-C, and GA designed the experiments. ÅG performed growing and sampling of plants, nutrient analyses, RNA extractions, and statistical analyses. RR-C performed secondary metabolite analyses. GS performed bioinformatics and statistical analyses of transcriptome data. ÅG wrote the first draft of the manuscript. All authors contributed to the article and approved the submitted version.

## Funding

This work was funded by the European Union’s Horizon 2020 research and innovation program under grant agreement no. 635727 Protein2Food.

## Conflict of Interest

The authors declare that the research was conducted in the absence of any commercial or financial relationships that could be construed as a potential conflict of interest.

## Publisher’s Note

All claims expressed in this article are solely those of the authors and do not necessarily represent those of their affiliated organizations, or those of the publisher, the editors and the reviewers. Any product that may be evaluated in this article, or claim that may be made by its manufacturer, is not guaranteed or endorsed by the publisher.

## Footnotes

^1^ http://planttfdb.gao-lab.org/

^2^ http://itak.feilab.net

^3^ https://rdrr.io/bioc/clusterProfiler/man/enrichGO.html

^4^ https://bioconductor.org/packages/release/bioc/html/clusterProfiler.html

^5^ https://rdrr.io/bioc/clusterProfiler/man/gseKEGG.html

^6^ https://gatk.broadinstitute.org

^7^ http://snpeff.sourceforge.net/SnpEff.html

^8^ https://m.ensembl.org/info/genome/variation/prediction/predicted_data.html

^9^ https://www.ncbi.nlm.nih.gov/genome/12754?genome_assembly_id=278928

## Supplementary Material

Supplementary Figure S1 Dry matter content (% by fw) of developing quinoa seeds of Titicaca, Pasankalla, and Regalona. For corresponding days post anthesis for each developmental stage, see the text. Results are showing mean values ± SD. Bars not sharing the same letter are significantly different according to Fisher’s pairwise test (*p* ≤ 0.05).

Supplementary Figure S2 Phenolic content **(A)**, flavonoid content **(B)**, and antioxidant capacity **(C)** in mature quinoa seeds on a dry weight basis. GAE, gallic acid equivalents and CE, cathequin equivalents. Antioxidant capacity is given as μmol trolox/100 g. Results are showing mean values ± SD. Bars not sharing the same letter are significantly different according to Tukey’s test (*p* ≤ 0.05).

Supplementary Figure S3 Results from KEGG pathway enrichment analysis based on the list of 3,568 differentially expressed transcripts (DETs) in Pasankalla as compared to the other genotypes.

Supplementary Table S1 Total number of processed reads from all raw samples from quinoa seed transcriptomes, aligned to the reference genome and used for abundance estimation. Samples were from early and midseed developmental stages (for corresponding days post anthesis see the text).

Supplementary Table S2 List of the 3,072 differentially expressed unique transcripts (from Table 1) in quinoa seeds from the comparison of Pasankalla as compared to Regalona and Titicaca (based on cutoff FDR<1e-2 and log2FC set to 1). The table contains the expression values (estimated raw read counts) in three biological replicates, log2fold changes, and the functional annotations of transcripts.

Supplementary Table S3 List of the 999 transcripts in quinoa seeds showing at least eight times higher/lower expression from the comparison of Pasankalla as compared to Regalona and Titicaca with the expression values (estimated raw read counts) in biological replicates, log2fold changes, and the functional annotations of transcripts. Of these, 60 transcripts were encoding either transcription factors, functions involved in central carbon metabolism of starch, sugar, protein and oil, or genes known to regulate embryo size which can be found in a separate folder.

Supplementary Table S4 Gene ontology (GO) term enrichment analysis of the 3,568 DETs found to be differentially expressed in Pasankalla as compared to Regalona and Titicaca. The 60 candidate genes (Figure 8) could be associated to 24 of the 48 GO terms listed and are marked in yellow.

Supplementary Table S5 List of the 2,863 SNPs/INDELs of high to moderate impact variants identified in the 3,072 DETs found in Pasankalla as compared to in Titicaca and Regalona. Of these SNPs/INDELs, the ones localized to the 60 selected target transcripts are shown in a separate folder.

Supplementary Table S6 List of all differentially expressed transcripts (DETs) (from Table 1) in quinoa seeds from all one-against-two and pair-wise genotype comparisons. The table contains the expression values (estimated raw read counts) in three biological replicates, log2fold changes (with cutoff FDR<1e-2 and log2FC is set to 1), and the functional annotations of transcripts.
