# Supplementary material for: Transcriptional Regulation of Quinoa Seed Quality: Identification of Novel Candidate Genetic Markers for Increased Protein Content
Source: Front Plant Sci. 2022 Jun 2;13:816425. doi: 10.3389/fpls.2022.816425 (PMC9201758; doi:10.3389/fpls.2022.816425)
Supplement: Supplementary Figure S1 — Dry matter content (% by fw) of developing quinoa seeds of Titicaca, Pasankalla, and Regalona. For corresponding days post anthesis for each developmental stage, see the text. Results are showing mean values ± SD. Bars not sharing the same letter are significantly different according to Fisher’s pairwise test (p ≤ 0.05). [file Data_Sheet_1.zip › Supplementary Data Sheet 1/Supplementary Table 1.pdf]

Quinoa genotype	Dev stage	Sequenced reads	Filtered number of reads	Uniquely mapped reads number	Uniquely mapped reads to Quinoa Genome (%)
Pasankalla	early	59244956	51439611	48958532	95.18%
	early	58572213	50683471	48180001	95.06%
	early	59588541	51715813	49099781	94.94%
	mid	71917080	59650500	56163887	94.15%
	mid	70354680	5777598	5396120	93.40%
	mid	67128816	50073573	46708957	93.28%
Regalona	early	47306009	40934972	38787546	94.75%
	early	6459075+ 44127876	43959982	41574703	94.57%
	early	1955181+ 30432792	26795106	25228125	94.15%
	mid	1431425+ 53433084	4952057	4721320	95.34%
	mid	23022370	19637795	18672776	95.09%
	mid	41667823	35714372	33996841	95.19%
Titicaca	early	54955968	44848257	42530226	94.83%
	early	6869592+ 33764850	34526720	32624481	94.49%
	early	65140284	49339112	46085003	93.40%
	mid	32176708	26136657	24664615	94.37%
	mid	33896020	25479910	23801287	93.41%
	mid	11489113+ 28007563	34302325	32601737	95.04%
