# Supplementary figures and images for: Transcriptional Regulation of Quinoa Seed Quality: Identification of Novel Candidate Genetic Markers for Increased Protein Content
Source: Front Plant Sci. 2022 Jun 2;13:816425. doi: 10.3389/fpls.2022.816425 (PMC9201758; doi:10.3389/fpls.2022.816425)

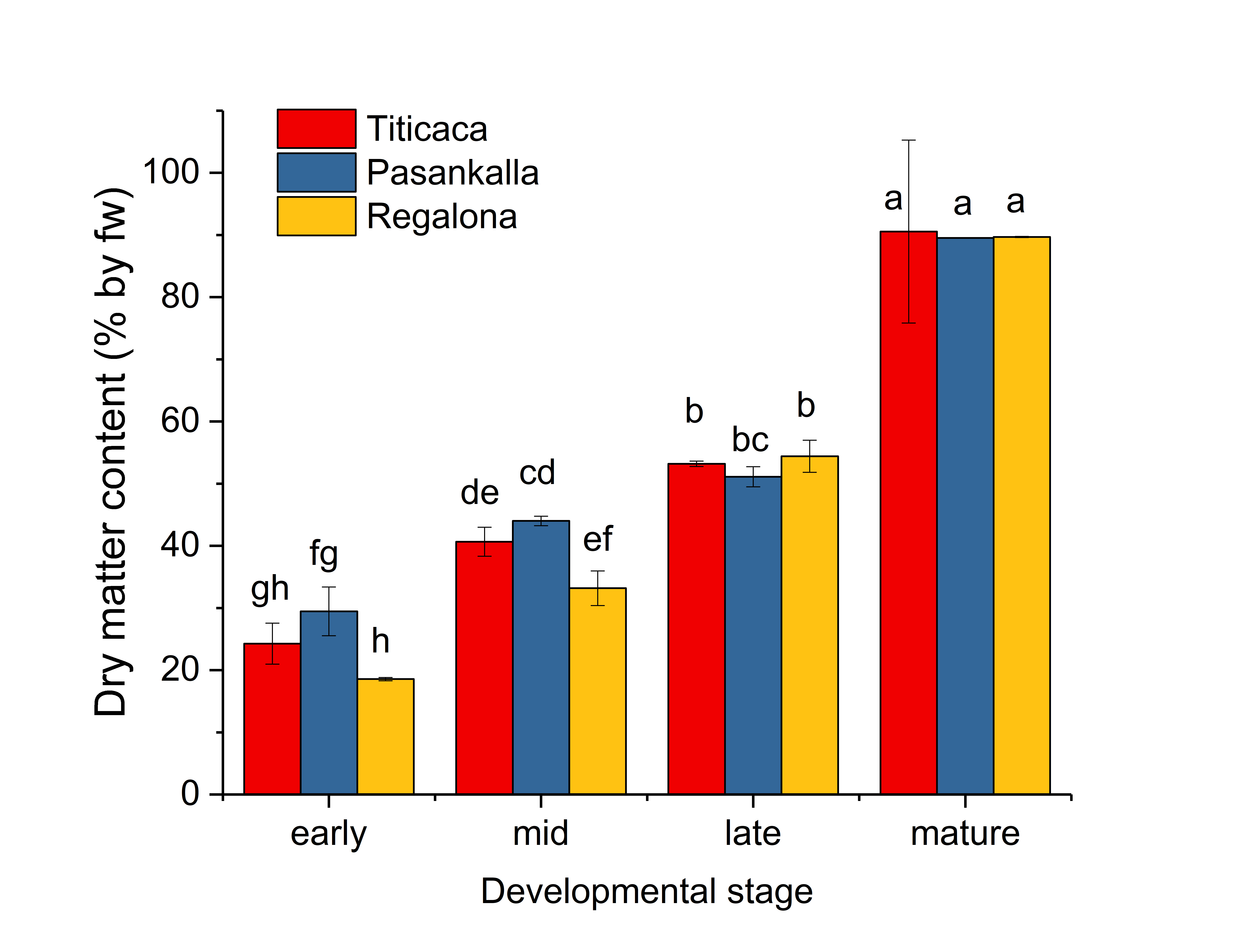

Supplement: Supplementary Figure S1 — Dry matter content (% by fw) of developing quinoa seeds of Titicaca, Pasankalla, and Regalona. For corresponding days post anthesis for each developmental stage, see the text. Results are showing mean values ± SD. Bars not sharing the same letter are significantly different according to Fisher’s pairwise test (p ≤ 0.05). [file Data_Sheet_1.zip › Supplementary Data Sheet 1/Supplementary Figure 1 .JPEG]

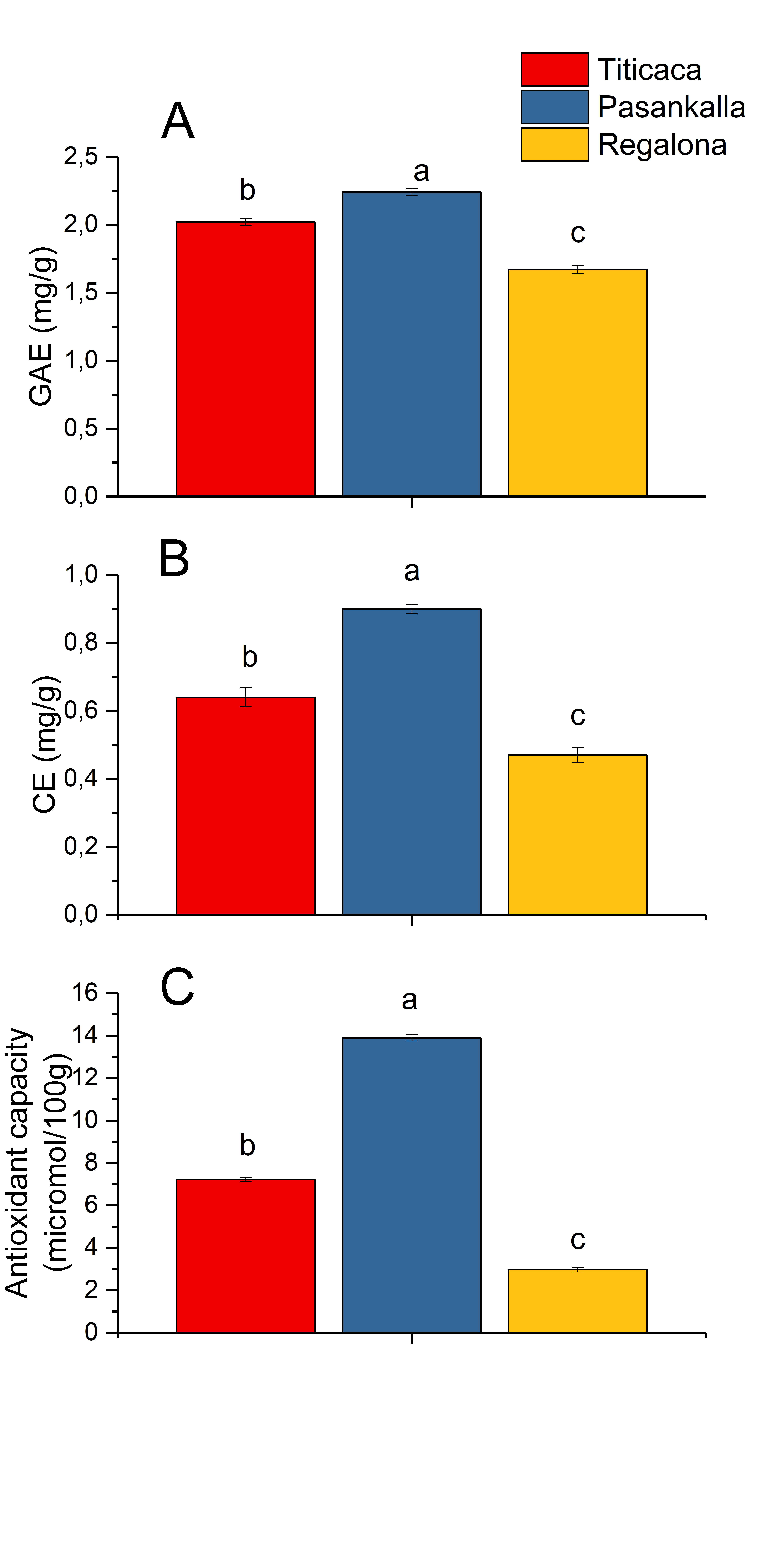

Supplement: Supplementary Figure S1 — Dry matter content (% by fw) of developing quinoa seeds of Titicaca, Pasankalla, and Regalona. For corresponding days post anthesis for each developmental stage, see the text. Results are showing mean values ± SD. Bars not sharing the same letter are significantly different according to Fisher’s pairwise test (p ≤ 0.05). [file Data_Sheet_1.zip › Supplementary Data Sheet 1/Supplementary Figure 2 .JPEG]

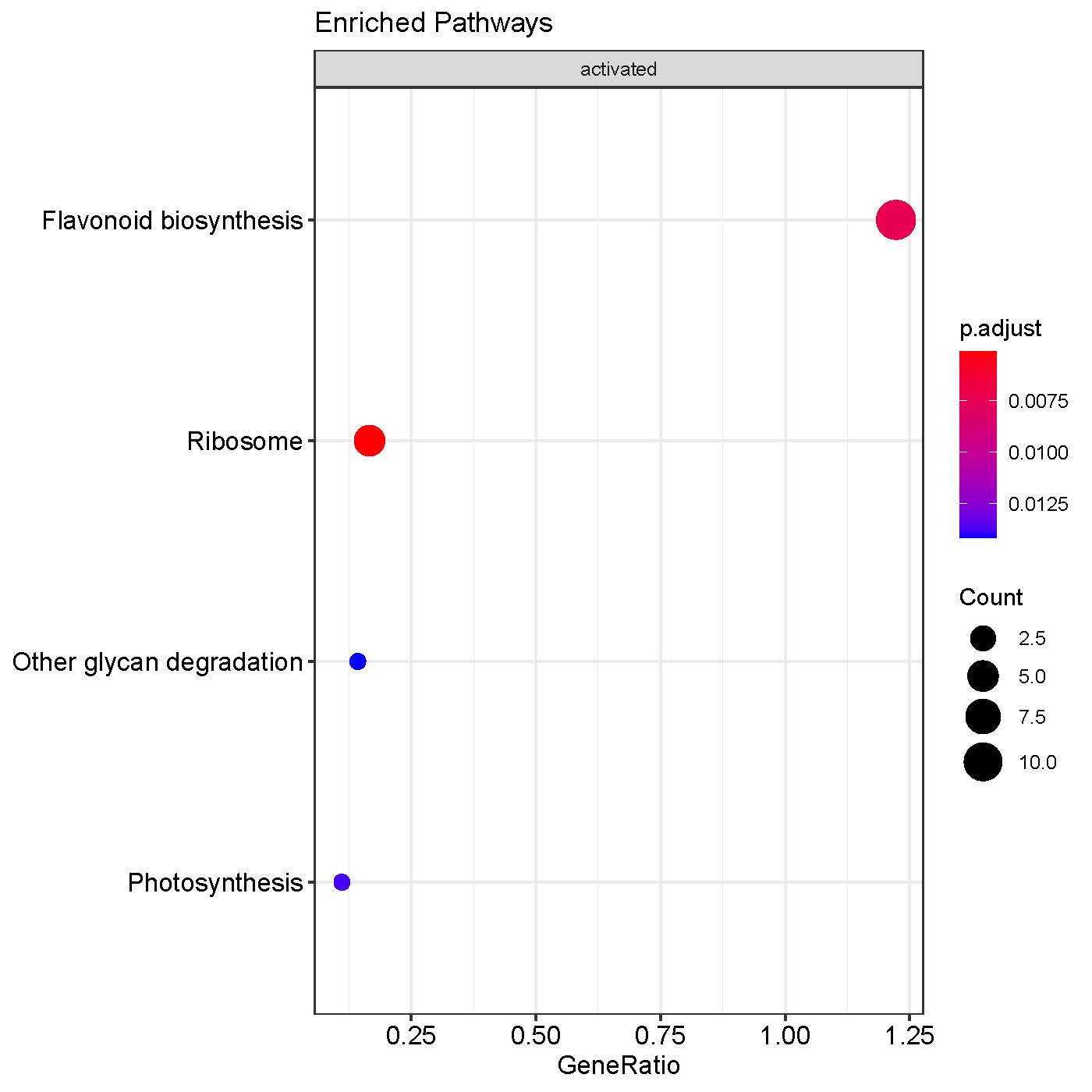

Supplement: Supplementary Figure S1 — Dry matter content (% by fw) of developing quinoa seeds of Titicaca, Pasankalla, and Regalona. For corresponding days post anthesis for each developmental stage, see the text. Results are showing mean values ± SD. Bars not sharing the same letter are significantly different according to Fisher’s pairwise test (p ≤ 0.05). [file Data_Sheet_1.zip › Supplementary Data Sheet 1/Supplementary Figure 3 .JPEG]
